# Mechanical stress and anionic lipids synergistically stabilize an atypical structure of the angiotensin II type 1 receptor (AT1)

**DOI:** 10.1371/journal.pcbi.1012559

**Published:** 2024-11-13

**Authors:** Rym Ben Boubaker, Daniel Henrion, Marie Chabbert

**Affiliations:** UMR CNRS 6015 –INSERM 1083, Laboratoire MITOVASC, Université d’Angers, Angers, France; Weizmann Institute of Science, ISRAEL

## Abstract

Environmental factors, including mechanical stress and surrounding lipids, can influence the response of GPCRs, such as the mechanosensitive angiotensin II type 1 receptor (AT1). To investigate the impact of these factors on AT1 activation, we developed a steered molecular dynamics simulations protocol based on quaternion formalism. In this protocol, a pulling force was applied to the N-terminus of transmembrane helix 6 (TM6) to induce the TM6 opening characteristic of activation. Subsequently, the simulations were continued without constraints to allow the receptor to relax around the novel TM6 conformation under different conditions. We analyzed the responses of AT1 to membrane stretching, modeled by applying surface tension, in different bilayers. In phosphocholine bilayers without surface tension, we could observe a transient atypical structure of AT1, with an outward TM7 conformation, at the beginning of the activation process. This atypical structure then evolved toward a pre-active structure with outward TM6 and inward TM7. Strikingly, the presence of anionic phosphoglycerol lipids and application of surface tension synergistically favored the atypical structure, which led to an increase in the cross-section area of the receptor intracellular domain. Lipid internalization and H-bonds between lipid heads and the receptor C-terminus increased in phosphoglycerol vs phosphocholine bilayers, but did not depend on surface tension. The difference in the cross-section area of the atypical and pre-active conformations makes the conformational transition sensitive to lateral pressure, and favors the atypical conformation upon surface tension. Anionic lipids act as allosteric modulators of the conformational transition, by stabilizing the atypical conformation. These findings contribute to decipher the mechanisms underlying AT1 activation, highlighting the influence of environmental factors on GPCR responses. Moreover, our results reveal the existence of intermediary conformations that depend on receptor environment and could be targeted in drug design efforts.

## Author summary

Mechanical forces play a critical role in the physiopathology of the cardiovascular system. The angiotensin II type 1 receptor (AT1) is a key regulator of cardiovascular functions. This receptor acts as a mechanosensor, capable of being activated by mechanical forces, although the underlying mechanism remains poorly understood. In this study, we investigate the relationship between environmental conditions, specifically mechanical stress and surrounding lipids, and the activation mechanism of AT1. To gain a deeper understanding of this complex interplay, we utilize steered molecular dynamics simulations based on quaternion formalism. Our study reveals an atypical conformation of the AT1 receptor which serves as a transient intermediate in the receptor activation pathway. This atypical conformation of AT1 is stabilized by a compelling synergy between mechanical stress (represented by surface tension) and anionic lipids (represented by phosphoglycerol lipids). This finding strongly suggests that specific lipids may play a crucial role in transmitting mechanical signals to the AT1 receptor. These insights will contribute to understand the development of diseases such as hypertension, where AT1 signaling is impaired in response to mechanical stress, and their link with the lipidome of the cardiovascular cell membranes. They can also help drug design targeted toward distinct conformations of AT1 to induce specific pathways.

## Introduction

G protein-coupled receptors (GPCRs) constitute the largest transmembrane receptor family in the human genome, with around 800 members, and contribute to most physiopathological functions. Upon binding of extracellular cognate ligands, they undergo conformational changes leading to binding and subsequent activation of effectors (usually G proteins and/or β-arrestins) which in turn induce cellular response [1]. Recent years have seen huge improvements in our understanding of GPCR mechanisms of action, with the multiplication of crystallographic and cryo-electromicroscopic structures [2–4]. These advances allow a deeper understanding of the complexity of GPCR regulation. Activation of a GPCR may lead to different responses and depends not only on the presence of an agonist but also on the presence of allosteric modulators or of physico-chemical external factors [1,5].

Several GPCRs are sensitive to mechanical stress [6,7]. This effect might be crucial in the cardiovascular system with permanent mechanical forces that are generated by heart contraction and blood flow, and include pressure, shear stress and stretch. These mechanical forces contribute to development, physiology and pathology, and involve a variety of mechanosensors that activate intracellular pathways [8–11]. Membrane stretching plays a crucial role in the heart physiology, regulating cardiac contractibility, cellular growth and remodeling. In arteries, along with shear stress, membrane stretching is crucial to regulate blood flow and myogenetic tone, in order to maintain blood pressure in physiological ranges. Impairment of these mechanisms is at the origin of cardiovascular diseases. The nature of surrounding lipids may also affect GPCR functions and oligomerization, either directly by specific interactions, or indirectly by modification of the environment fluidity [12,13]. For example, studies based on experimental approaches [14,15] or molecular dynamics (MD) simulations [16,17] report that anionic lipids maintain or favor the active state of different receptors. This may affect GPCR responses in various cardiovascular cell types such as cardiomyocytes, endothelium, and vascular smooth muscle cells, which contain anionic lipids [18–21]. Moreover, cardiovascular pathologies are associated with important lipidome alterations which affect these cell membranes [18–21]. Understanding the influence of anionic lipids on the activity of cardiovascular GPCRs is thus an important step to decipher changes in GPCR responses in cardiovascular diseases.

The angiotensin II type 1 receptor (AT1) is an example of mechanosensitive receptor involved in the cardiovascular system. It is able to detect membrane stretching or shear stress, and might be activated by mechanical stress alone [22–30]. A recent MD study indicates that the active state of AT1 is sensitive to membrane thickness and tension [31]. In our study, we aimed to investigate the effect of external factors (lipids and mechanical stress) on the *activation mechanism* of AT1. In particular, we aimed to determine whether, in model systems, specific lipids may facilitate receptor response to mechanical stress in the absence of an agonist.

The microsecond timescale that we can explore upon classical MD simulations is not sufficient to observe GPCR activation-like conformational transitions. Observation of these transitions requires specific techniques such as accelerated MD simulations [32,33], metadynamics [34], adaptive biasing [35] or steered methods [36,37]. The latter methods are based on the *a priori* knowledge of initial inactive and final active states and on an applied force to a collective variable that helps the system to overcome the transition barrier between the two conformations [35–37].

Since the resolution of the β2-adrenergic receptor in complex with Gs [38], canonical activation of GPCRs is described by two major changes: an outward motion of transmembrane helix 6 (TM6) and an inward motion of transmembrane helix 7 (TM7). Nevertheless, comparison of active structures in complex with various G proteins or with β-arrestins indicates that different active states exist [1–3]. In addition, alternative, non-canonical structures combining features of active and inactive conformations have been reported [39], suggesting a complex activation pathway with intermediate conformations. Both the canonical and non-canonical active structures are characterized by an outward motion of TM6 that opens the intracellular cleft for effector binding, but they differ by the canonical “inward” and non-canonical “outward” position of TM7, by reference to the inactive state.

AT1 is a prototype of biased receptor. Distinct conformations of this receptor have been reported by crystal structure resolution [40,41], biophysical studies [42] and molecular dynamics simulations [31,43,44]. Here, to gain insights into the activation process and the influence of external factors on this process, we searched a steered molecular dynamics (SMD) method that uncouples the motions of TM6 and TM7. The TM6 opening motion can be described either by the Euler bending and wobbling angles [45] or by a quaternion describing the angle and the axis of the motion [46]. Thus, the steered molecular dynamics method based on the quaternion formalism imposes a constraint on TM6 but leaves the other helices free. We used this method to compare the steps leading to the activation of the human AT1 receptor under different environmental conditions, which combines bilayer composition and mechanical stress. Mechanical stress was modeled by application of surface tension (equivalent to negative lateral pressure) to the Newton equations of movement, which results in membrane stretching. Our results reveal a synergy between anionic lipids and surface tension which stabilizes an atypical conformation of AT1.

## Results

### Preliminary classical molecular dynamics simulations

To investigate the impact of environmental conditions on the conformational transition of the AT1 receptor, we performed preliminary classical MD simulations (cMD), which were conducted for a duration of 220 ns. These simulations involved the human AT1 receptor embedded in diverse phospholipid bilayers, under two distinct conditions: NPT conditions (constant number of particles, pressure and temperature) and NPγT conditions (constant number of particles, pressure, surface tension, and temperature) with a surface tension γ applied to mimic membrane stretching. In our simulations, we set the surface tension γ to a value of 20 dyn/cm. This value was chosen because it induces a 10% stretch in lipid area (see below), which matches the 10% cyclic mechanical stretch yielding mechanoactivation of the AT1 receptor [27]. This value is also consistent with previous experimental data and simulations conducted on mechanosensitive proteins [47,48].

**Fig 1:**
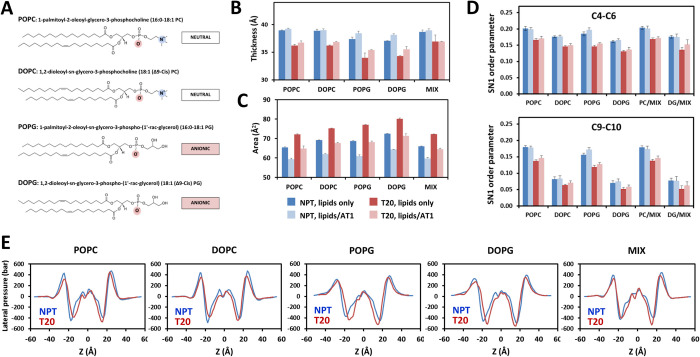
Lipid properties. (A) Chemical formula and full names of POPC, DOPC, POPG and DOPG, highlighting the differences in the heads, tails and charges of the lipids under scrutiny. Adapted from https://avantilipids.com; (B-D) Lipid physical parameters during classical MD simulations carried out without (NPT) or with (T20) applied surface tension of 20 dyn/cm (blue and red bars, respectively), in the absence or presence of embedded AT1 (dark and light colors, respectively). Data are the average ± standard deviation from three cMD trajectories. The bilayer thickness is in (B). The lipid area is in (C). The summary of the order parameters *S*_*CH*_ for the carbon atoms from the SN1 chain is in (D). The average *S*_*CH*_ for carbons C4 to C6 (maximum value) and for carbons C9 and C10 (different in saturated and unsaturated lipids) are in the top and bottom panels, respectively; (E) Lateral pressure profiles *π(z)* measured along the Z axis, defined by the normal to the bilayer plane, for each indicated bilayer with embedded AT1, without (NPT) or with (T20) applied surface tension of 20 dyn/cm. The receptor is oriented along the Z axis with positive values toward the intracellular side.

We selected five bilayer compositions (see the chemical formula in Fig 1A). Two bilayers were composed of zwitterionic phospholipids, either the 1-palmitoyl-2-oleoyl-glycero-3-phosphocholine (POPC) or the 1,2-dioleoyl-sn-glycero-3-phosphocholine (DOPC). Two bilayers were composed of anionic phospholipids, either the 1-palmitoyl-2-oleoyl-sn-glycero-3-phosphoglycerol (POPG) or the 1,2-dioleoyl-sn-glycero-3-phosphoglycerol (DOPG). Finally, the fifth bilayer was a mixture of POPC and DOPG with a 9:1 ratio (MIX) to mimic more physiological conditions. We also performed control simulations of the same hydrated bilayers in the absence of embedded receptor to analyze the effect of AT1 on the properties of the lipids. The effects of the environmental conditions on the lipid physical parameters are reported in Figs 1 and S1. Data are consistent with previous reports on DOPC [49,50] and POPC [51] parameters, surface tension effects [50–53] and protein insertion effects [51,54].

Briefly, the DOPG and POPG thickness slightly increased by about 1 Å with AT1 insertion (Fig 1B). In the presence of AT1, the average thickness for the lipids under scrutiny was 38.8 ± 0.5 Å in NPT conditions, and decreased by about 6% to 36.3 ± 0.8 Å, under a surface tension of 20 dyn/cm. Both in NPT and NPγT conditions, embedded AT1 receptor induced a decrease in the area per lipid of about 10% (Fig 1C), which may be related to interface effects between the lipids and the receptor [54]. A surface tension of 20 dyn/cm increased the area per lipid by about 10%, both in the presence and in the absence of AT1. As a result, the lipid areas for the lipids alone were similar to those observed under a surface tension of 20 dyn/cm with embedded receptor. The differences in the order parameters of the SN1 carbons in 1-palmitoyl and 1-oleoyl lipids (Figs 1D and S1) result from the saturation of the SN1 chain in 1-palmitoyl lipids compared to 1-oleoyl lipids, which markedly decreases the chain fluidity (0.17 ± 0.1 and 0.08 ± 0.1, respectively, at the double bond position). Whatever the lipid, application of the surface tension induced a decrease in the order parameters of about 17% for each SN1 and SN2 position (see S1 Fig for full data set), in agreement with DOPC data [50].

We also measured the lateral pressure profile *π(z)* (see the Methods Section for details) along the Z axis normal to the bilayer plane, for each lipid environment with embedded AT1, without and with an applied surface tension of 20 dyn/cm (Fig 1E). These profiles are similar to other reports [49,55,56]. They are characterized by two strong positive peaks, corresponding to phospholipid heads, and two strong negative peaks, corresponding to the polar-apolar interface (interfacial tension) [57]. The lateral pressure within the core of the bilayer is close to 0, due to the spool shape of the receptor. Membrane stretching due to an applied surface tension induces a contraction of the pressure profile which brings closer the positive and negative peaks by 2.4 ± 0.3 Å and 4.2 ± 1.0 Å, respectively, in link with the reduced bilayer thickness. Notably, the positive peaks are wider in phosphoglycerol than phosphocholine bilayers, which may result from the repulsion between anionic heads and/or increased interactions with the receptor (see below).

In contrast to the significant impact of surface tension on the lipid properties, neither surface tension nor the lipid environment affected the stability and conformation of the inactive AT1 receptor, which could be measured by the distances between the TM3-TM6 and TM3-TM7 helices and by the cross-section areas of the AT1 receptor during classical MD simulations (see data in S2 Fig).

### Quaternion based steered molecular dynamics simulations of the AT1 receptor

**Fig 2:**
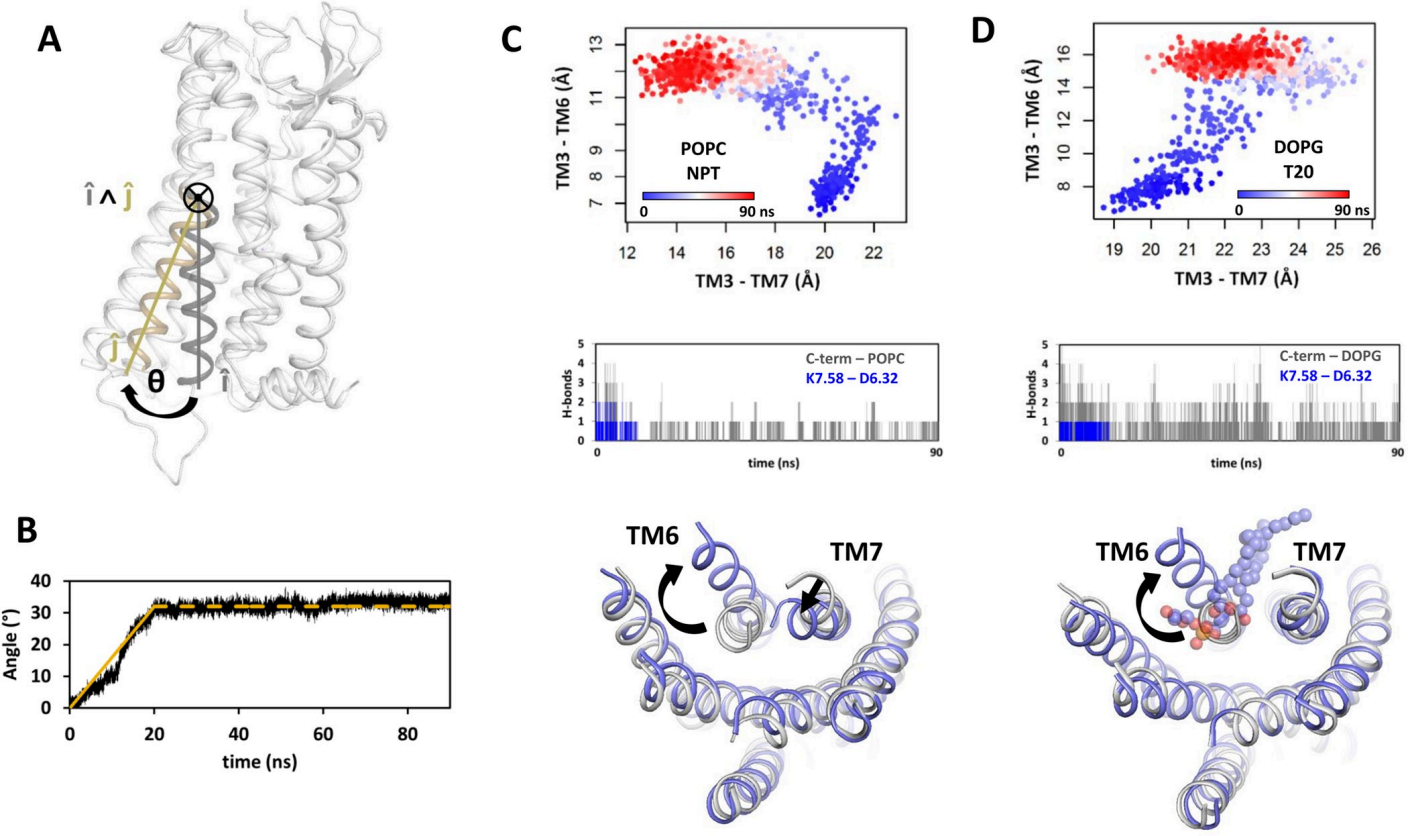
Overview of the method and typical results of the quaternion-based steered MD simulations of receptor activation. (A) Schematic representation of the quaternion describing the TM6 motion. The inactive and active structures of AT1 are superimposed. The rotation axis of the TM6 N-terminal half is obtained from the cross product of the vectors representing the axis of TM6 before (î, silver) and after (ĵ, golden) the transition; (B) Typical evolution of the angle θ describing the motion of TM6 during the steered MD simulation. The pulling force was applied to open the θ angle to 35° in a timeframe of 20 ns (32° observed, continuous golden line), and then the simulation proceeded for 70 ns (dashed line); (C, D) Examples of steered MD simulations of AT1 in POPC under NPT conditions (C) and in DOPG under NPγT conditions with γ = 20 dyn/cm (D). The top panels are 2D plots of the conformational transitions, showing the TM3-TM6 distance versus the TM3-TM7 distance during the simulation, with time indicated by the color of the dots (from blue to red, representing the progression of time over a 90 ns timeframe). The central panels represent stacked plots of the number of H-bonds between D6.32 and K7.58, in blue, and of the total number of the H-bonds between the lipids and the positive residues in the AT1 C-terminus (K7.58, K7.59, K7.61, R7.62), in grey. The bottom panels are superimposed ribbon representations of the AT1 transmembrane helix bundle at the beginning (grey) and at the end of the simulations (slate). In (D), an internal DOPG molecule is represented as spheres with oxygen and phosphorus atoms in red and orange, respectively.

The lack of environmental effects on the inactive receptor conformation prompted us to investigate the influence of the environment on the receptor activation process. To do that, we developed a steered MD simulations protocol to induce a conformational transition in the receptor (see the Methods Section for details). This method was based on the opening motion of the N-terminal half of TM6 which is a prominent characteristic of the activation transition [38]. The pulling force was based on the quaternion associated with the rotational motion of the N-terminal half of TM6 upon activation (Fig 2A). Following the equilibration of the system during a preliminary cMD step, a pulling force was applied on the N-terminal half of TM6 for a duration of 20 ns. Subsequently, the simulations continued without constraints for 70 ns to allow the system to relax. The opening of TM6, monitored by the rotational angle θ, accompanied the pulling force, and reached the target angle at the end of the 20 ns steering procedure. A typical example of the evolution of the angle θ during the simulations is presented in Fig 2B. Thereafter, we will use the terms “steered molecular dynamics” or “SMD” to refer to the complete protocol, which includes both the pulling step and the relaxation step.

This procedure presented three advantages:

1. Both the bending and wobbling motions of TM6 upon the transition were taken into account, in a straightforward way;
2. The TM6 structure was not constrained as a rigid body but remained flexible during the conformational transition. Twisting and wriggling were similar to those previously observed in GPCR activation obtained by accelerated MD simulations [33] and might contribute to lower the activation barrier;
3. No constraint was applied on TM7, suggesting that conformational changes observed in TM7 might correspond to the structural responses of the receptor to adapt to the TM6 reorientation.

### Conformational changes of AT1 in two representative conditions

For clarity purpose, we will detail the conformational changes of AT1 in two representative trajectories, yielding different final conformations, and then we will analyze how the environmental conditions favor each conformation. Fig 2 displays typical 2D graphs of the AT1 transition, in which the motions of both TM6 and TM7 (quantified by the TM3-TM6 and TM3-TM7 distances) were monitored simultaneously. In Fig 2C, AT1 was embedded in a POPC bilayer under NPT conditions and underwent a transition toward a pre-active conformation, with outward TM6 and inward TM7. The transition was biphasic. In the first step, the TM6 motion was accompanied by a slight outward motion of TM7, up to a TM6 opening angle of about 15–20°. These correlated motions could be related to H-bonds between D6.32 in TM6 and K7.58 in the TM7-H8 kink which were broken when the θ angle reached a threshold (Fig 2C, central panel). In the second step, the two helices moved in opposite directions. TM6 continued with an outward motion whereas TM7 underwent an inward motion. The completion of the inward motion of TM7 occurred during the relaxation step that followed the pulling step. The final structure with outward TM6 and inward TM7 was reached within the 90 ns timeframe of the simulation.

The behavior of AT1 was strikingly different in anionic DOPG bilayer, under a surface tension of 20 dyn/cm (Fig 2D). In this case, we observed an initial opening of both TM6 and TM7. However, after the H-bonds between TM6 and TM7 were broken, we did not observe an inward motion of TM7, despite the outward motion of TM6. The TM3-TM7 distance transiently increased up to 25 Å and was stabilized around 22 Å at the end of the simulation. The open position of TM7 was maintained by H-bonds between the receptor C-terminus and the lipid heads (Fig 2D, central panel). These H-bonds contributed to the opening of TM7 up to a distance of 25 Å, which allowed the ingress of a DOPG molecule within the receptor intracellular cavity. The internal DOPG prevented any subsequent inward motion of TM7. Internalization of a DOPG molecule has previously been reported during a microsecond long classical MD simulation of A2aR in a DOPG bilayer [16].

### Analysis of the AT1 conformational changes under diverse environmental conditions

**Fig 3:**
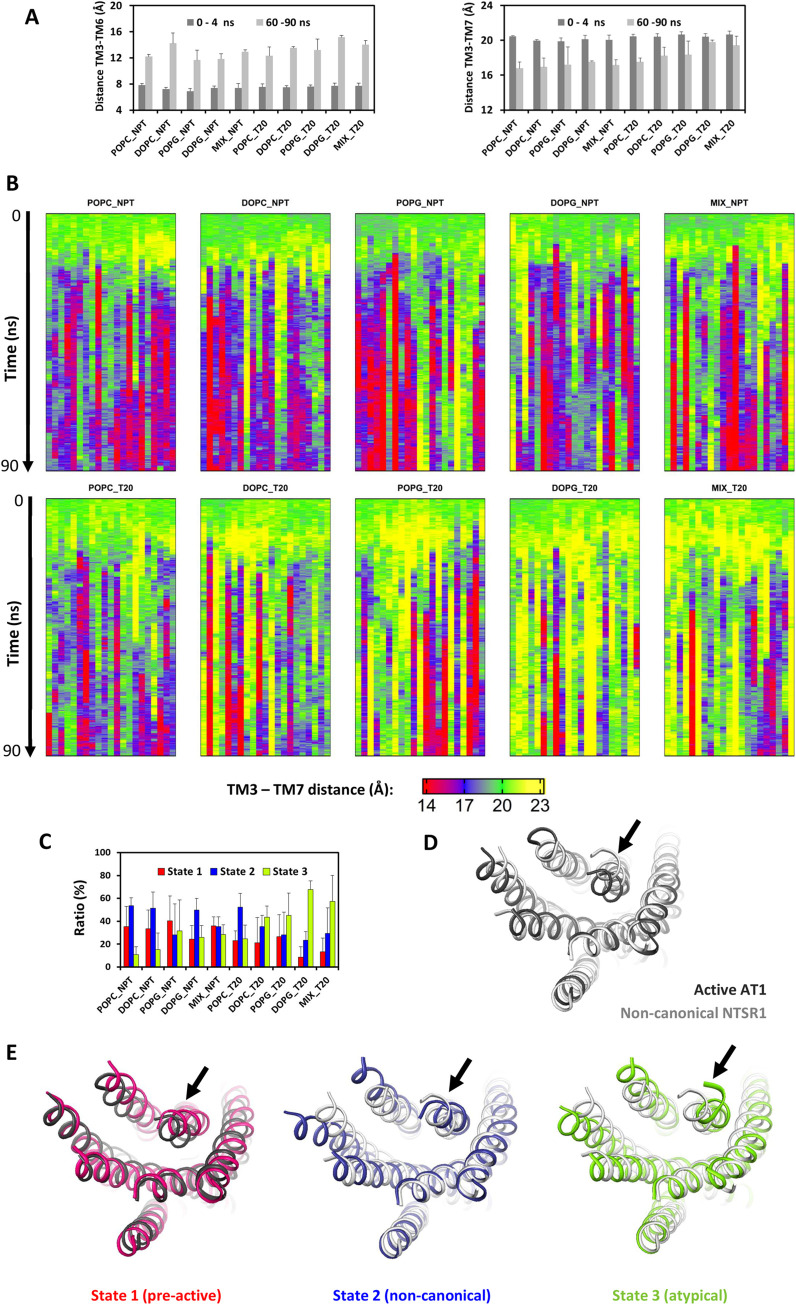
Conformational space screened by AT1 during steered MD simulations. (A) Average TM3-TM6 (left) and TM3-TM7 (right) distances at the beginning (dark grey, time 0 to 4 ns) and at the end (light grey, time 60 to 90 ns) of the SMD simulations, as a function of environmental conditions. Standard deviations were calculated from the means of the 7 replicas by initial snapshot; (B) Time evolution of the TM3-TM7 distance observed during the SMD simulations for each of the 21 replicas carried out in the indicated condition (7 replicas for 3 initial snapshots). The color code is red, blue, green and yellow for TM3-TM7 distances lower than 16 Å, between 16 and 19 Å, between 19 and 21 Å and larger than 21 Å. The average TM3-TM7 distance in the initial snapshots was 20 ± 1 Å (green); (C) Summary of the conformations reached during the last 30 ns of the simulations on the basis of the TM3-TM7 distance. The color code is red, blue and lime for TM3-TM7 distances lower than 16 Å (pre-active sate), between 16 and 19 Å (non-canonical state) and larger than 19 Å (atypical state). In (A-C), the simulations were carried out in the indicated bilayer, without (NPT) or with (T20) an applied surface tension of 20 dyn/cm. The MIX bilayer corresponds to a POPC/DOPG mixture in the ratio of 9 to 1. The total duration of the simulations was 90 ns. The pulling force to open TM6 was applied during the first 20 ns and then the simulations continued without constraints; (D) Superposition of the canonical active structure of AT1 in complex with angiotensin II and a stabilizing nanobody (PDB 6OS0, dark grey) and of the non-canonical active structure of the neurotensin receptor 1 (NTRS1) in complex with the agonist JMV449 and Gi (PDB 6OSA, light grey); (E) From left to right, ribbon visualization of representative final conformations of AT1 which are in the pre-active, non-canonical, and atypical states (red, blue and lime, respectively). The pre-active conformation was obtained in POPC under NPT conditions and is superposed on the active structure of AT1 (red and dark gey ribbons, respectively). The non-canonical (blue) and atypical (lime) conformations were obtained in DOPG under an applied tension of 20 dyn/cm and are superimposed on the non-canonical structure of NTSR1 (light grey ribbon). In these AT1 conformations, TM6 is open and the TM3-TM7 distances are 14.7, 17.0 and 23.2 Å, for the pre-active, non-canonical and atypical conformations, respectively. In (D, E), only the transmembrane helix bundles are shown for clarity purpose.

Ten environmental conditions were under scrutiny (five lipid environments, with or without added surface tension). To obtain representative sets of data, for each environmental condition, we selected three initial snapshots from equilibrated classical MD simulations carried out in the same conditions and we reiterated the simulations seven times for each initial snapshot (see Methods). As previously, the receptor conformation during the steered simulations was monitored by the TM3-TM6 and TM3-TM7 distances, and visualized with 2D graphs (S3 Fig) and heatmaps (Figs 3 and S4).

The steered TM6 opening was completed within 20–25 ns, but reached different final amplitudes, both between and within subsets (S4 Fig). Briefly, in the absence of tension, the opening was larger when AT1 was embedded in phosphatidylcholine rather than phosphoglycerol lipids. The reverse was observed under applied surface tension. The averaged TM3-TM6 distances during the final 30 ns ranged from 11.6 ± 1.5 Å, in POPG bilayer without surface tension, to 15.2 ± 0.3 Å, in DOPG bilayer under surface tension (Fig 3A).

These TM6 data are consistent with the diversity of TM6 positions that have been observed in the structures of active GPCRs obtained by X-ray or cryo-electron microscopy [2,3]. More surprising is the diversity of TM7 structures observed during our simulations, which span the spectrum between the two limits depicted in Fig 2. The TM7 motion could be visualized by heatmaps of the TM3-TM7 distances, with a four color code (Fig 3B). The green color indicates a TM3-TM7 distance similar to the inactive state (19–21 Å) whereas the yellow color indicates an outward motion of TM7 with TM3-TM7 distance larger than 21 Å. The blue and red colors indicate, respectively, a TM3-TM7 distance between 16 and 19 Å, and below 16 Å. Standard deviations of the final TM3-TM7 distances from the replicas carried out in the same conditions could reach ± 3 Å. Nevertheless, when we considered the standard deviations from the means by initial snapshot, we observed a clear trend in the final TM3-TM7 distance which increased from 16.8 ± 0.7 Å, in POPC bilayer without surface tension, to 19.8 ± 0.2 Å, in DOPG bilayer under surface tension (Fig 3A, right panel). These data support the assumption that the inward motion of TM7 is favored in neutral bilayers without surface tension whereas the outward position of TM7, similar to or more open than TM7 in the inactive state, is favored in the presence of anionic lipids under surface tension.

For a deeper analysis of the final TM7 conformation as a function of the environment, we utilized a three level classification based on the TM3-TM7 distances (Fig 3C). This classification matched the heatmap color code except that the green and yellow states, separated in the heatmaps for clarity purpose, were clustered into a single conformation (lime color in Fig 3C), characterized by TM3-TM7 distance equal to or larger than the distance in the inactive structures, 19 Å.

The AT1 conformations observed during the SMD simulations, with outward position of both TM6 and TM7, were reminiscent of the non-canonical (NC) structure of the neurotensin receptor 1 (NTSR1) in complex with Gi (PDB 6OSA) [39]. This structure differs from the canonical active receptor structure with inward TM7 position that is observed, for example, in the structure of active AT1 receptor in complex with angiotensin II and a stabilizing nanobody (PDB 6OS0) [41] (Fig 3D). Three conformations representative of each AT1 state were compared to these two structures (Fig 3E). The red conformation presented an inward motion of TM7 and could correspond to a pre-active conformation. The blue conformation had a TM7 position similar to the non-canonical structure of NTSR1, while the lime conformation was atypical with extreme opening of TM7.

Data in Fig 3 draw several observations:

1. Each trajectory depended on interplay of specific and general features, making reiteration mandatory to gain information on the influence of the environmental factors on the conformational transition of AT1.
2. A slight outward motion of TM7 was frequently observed at the beginning of the simulations, as displayed in Fig 2C. Inward motion was possible only after the breaking of the H-bonds between D6.32 and K7.58, if they were present at the beginning of the simulations.
3. In all conditions, the inward motion of TM7 was delayed as compared with the outward motion of TM6. Even in the most favorable cases (POPC/DOPC bilayer under NPT conditions), it initiated after the opening of TM6.
4. The ability of the AT1 receptor to reach the pre-active state (red color) depended of the environment. It was favored in neutral phospholipids (POPC, DOPC) under NPT conditions.
5. The conformation characterized by an extreme opening of TM7 (lime color) was predominantly observed when anionic lipids (POPG, DOPG and MIX) were present, along with applied surface tension. In the presence of both negative lipids and surface tension, this atypical conformation was observed in approximatively 65% of the replicas during the final 30 ns of the trajectories.

### Gross conformational changes of the AT1 receptor in diverse environments

**Fig 4:**
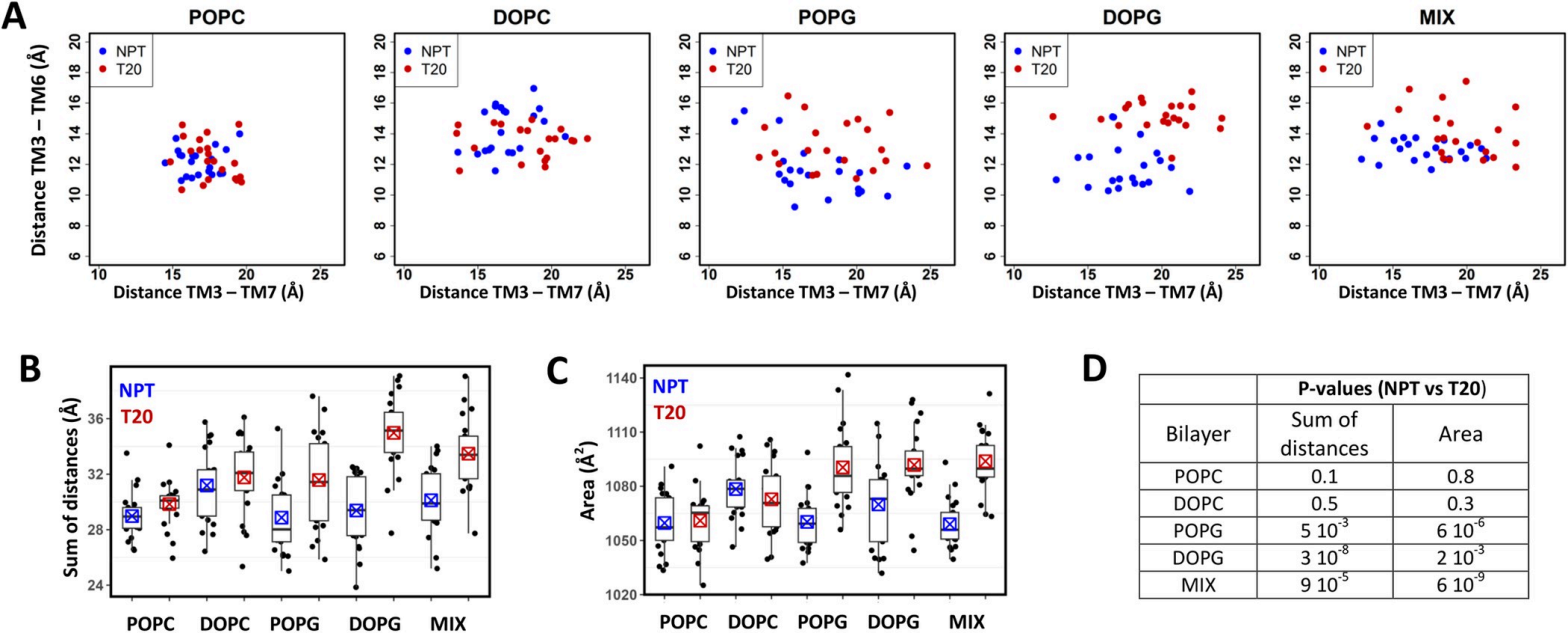
Gross conformational changes of the AT1 receptor under surface tension. (A) 2D-plots of the average TM3-TM6 distances versus the average TM3-TM7 distances measured for each replica during the final 30 ns of the SMD trajectories. The SMD simulations were performed in the indicated bilayers, without (NPT, blue dots) or with an applied surface tension of 20 dyn/cm (T20, red dots); (B) Boxplots of the sum of the TM3-TM6 and TM3-TM7 distances, as a collective variable of AT1 conformation, drawn from the data reported in (A), with the same color code (NPT, blue symbols; T20, red symbols); (C) Boxplots of the average cross-section area of the receptor moiety embedded into the intracellular bilayer leaflet (z ranging from 0 to 20 Å), measured for each replica during the last 30 ns of the trajectories, in the indicated bilayer, without (blue symbols) or with applied surface tension (red symbols). In (B, C), the boxes indicates the first and third quartile, the median is represented by the horizontal bold line, and the mean is represented by the boxed cross symbol; (D) Table giving the p-values indicating the probability of no significant difference (Null hypothesis) between data obtained with and without applied surface tension for each bilayer composition. P-values were calculated with the Student’s t-test.

We also investigated the global changes of the AT1 receptor in the diverse environments under scrutiny. Fig 4 displays the 2D graphs of the average TM3-TM6 distances versus the average TM3-TM7 distances observed for each replica during the final 30 ns of the trajectories. No change was observed upon application of surface tension in POPC and DOPC bilayers. This was not the case for POPG, DOPG and MIX bilayers, in which the conformation of AT1 under surface tension was characterized by an increase in both the TM3-TM6 and TM3-TM7 distances. Consequently, to describe the receptor conformation, we utilized the sum of the TM3-TM6 and TM3-TM7 distances, as a collective variable. We then examined the impact of surface tension on this collective variable for each lipid environment. No changes were observed in POPC and DOPC bilayers when surface tension was applied. However, in anionic bilayers, including the MIX bilayer, very significant changes were observed, with p-values ranging from 5 10^−3^ to 3 10^−8^.

To extend this analysis, we measured the cross-section areas of the AT1 receptor during the last 30 ns of the SMD simulations (see S5 Fig for full data set). Data are summarized in Fig 4C with boxplots of the average cross-section area of the receptor moiety embedded into the intracellular bilayer leaflet. When only neutral lipids were present, the application of surface tension did not induce any change in the cross-section of the receptor. However, in the case of anionic lipids, the application of surface tension led to a notable increase in the average cross-section areas (22–35 Å^2^). This increase was statistically significant, with p-value ranging from 2 10^−3^ to 6 10^−9^ (Fig 4D). Notably, the largest difference of 35 Å^2^ was observed in the MIX bilayer in which only 10% of lipids were anionic.

### Lipid interactions with the AT1 receptor in diverse environments

**Fig 5:**
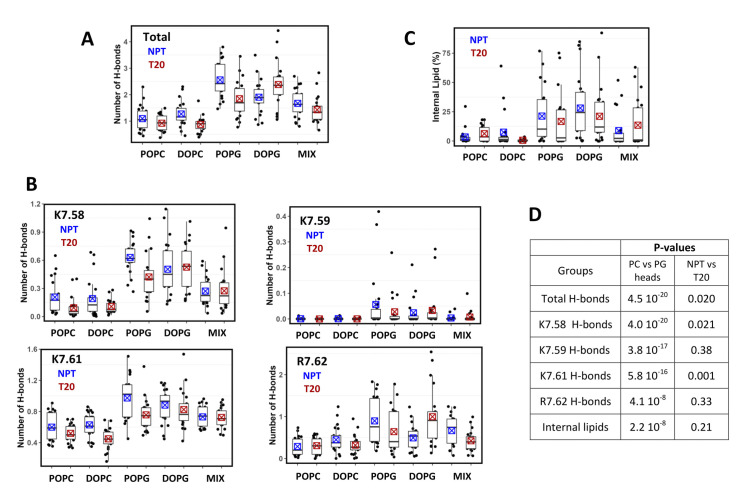
Interactions of the lipids with the AT1 receptor. (A, B) Boxplots of the average number of H-bonds between lipids and the receptor C-terminus (A) or between lipids and individual positions (K7.58, K7.59, K7.61 and R7.62) in the receptor C-terminus (B), observed during the last 30 ns of the SMD simulations for each replica in the different bilayers in the absence (NPT, blue symbols) or in the presence of an applied surface tension of 20 dyn/cm (T20, red symbols); (C) Boxplots of the ratio of frames with an internal lipid molecule, measured for each replica, in the different bilayers without (NPT) or with applied surface tension of 20 dyn/cm (T20); (D) Table giving the p-values indicating the probability of no significant difference (Null hypothesis) between groups resulting from pooling data obtained (1) in phosphatidylcholine versus phosphatidylglycerol bilayers or (2) in the absence versus the presence of an applied surface tension of 20 dyn/cm. P-values were calculated with the Wilcoxon test. In (A-C), the boxes indicate the first and third quartile, the medians are represented by the horizontal bold line, and the means are represented by the crossed square symbol.

To gain further information on factors favoring the different conformations of AT1, we analyzed the interactions of the lipids with the AT1 receptor with two criteria: (1) the H-bonding interactions between the positive charges of the receptor C-terminus and the lipid heads and (2) the lipid internalization within the receptor intracellular cavity. The C-terminus of AT1 possesses four positive charges (K7.58, K7.59, K7.61 and R7.62) that can interact with the lipid heads. Heatmaps of the total number of H-bonds between the receptor C-terminus and the lipids (S6 Fig) highlight the variability of H-bond interactions during the simulations timescale between replicas, for each condition under scrutiny. To take into account this variability, we report data as boxplots of the average number of H-bonds by replica, as a function of environmental conditions (Fig 5A). As expected, the H-bond interactions were significantly higher with phosphoglycerol (PG) than phosphocholine (PC) lipids. The total numbers of H-bonds were 2.2 ± 0.8 and 1.0 ± 0.4, for PG and PC lipids, respectively, when data were pooled independently of applied tension. In the POPC/DOPG bilayer (MIX), DOPG molecules were overrepresented in the vicinity of the receptor C-terminus, forming an average of 1.6 ± 0.5 H-bonds. We used the non-parametric Wilcoxon test for statistical analysis of pooled data. The p-value of 4.5 10^−20^ between phosphocholine and phosphoglycerol data highlights the significant difference in H-bonding interactions between these lipids. We also checked H-bonding pattern to specific residues of AT1 (Fig 5B). Briefly, interactions with K7.58 occurred mainly in PG bilayers (0.5 ± 0.2 H-bonds, compared to 0.15 ± 0.15 H-bonds in PC bilayers). Interaction with K7.59 was marginal except for a few replicas in phosphoglycerol bilayers. K7.61 was the position most favorable for interaction with PC lipids (0.9 ± 0.2 and 0.5 ± 0.2, for PG and PC lipids, respectively), whereas interaction with R7.62 was highly variable (0.8 ± 0.6 and 0.3 ± 0.2, with PG and PC, respectively). The increase in H-bond interactions in the presence of anionic lipids was significant for all individual positions (p-values ranging from 4 10^−8^ to 4 10^−20^).

We also compared data on the basis of the applied tension, but we did not observe a general rule. In most cases, we observed a trend to a decrease in H-bonds (especially for POPG) or no changes when a surface tension of 20 dyn/cm was applied, as compared to NPT conditions (p-value ranging from 0.001 to 0.3). Nevertheless, DOPG did not fit this pattern, with an increase in total interactions (mainly due to R7.62) upon applied tension. We cannot rule out specific effects due to either the initial conditions or the nature of the lipid, but, in any case, these observations preclude conclusion on the effect of applied tension on H-bonding interactions.

We also examined the extent to which lipid molecules could penetrate the intracellular cavity of the AT1 receptor, as illustrated in Fig 2D. Visual inspection of the trajectories indicated that lipid internalization occurred through either the TM5-TM6 or the TM6-TM7-H8 clefts. Noteworthy, lipid molecules were not trapped and could exit, especially when they remained in the vicinity of the clefts. Consequently, for each trajectory, we determined the ratio of frames with internal lipid heads and represented the data with boxplots (Fig 5C). In both NPT and NPγT conditions, stable penetration of lipids was found to be marginal in POPC and DOPC bilayers. However, in bilayers with anionic lipids (POPG, DOPG and MIX), the incidence of lipid penetration increased significantly, occurring in about 50% of the trajectories. Pooling data to compare results obtained in phosphoglycerol and phosphocholine bilayers led to a p-value of 2 10^−8^. Pooling data to compare results obtained with and without surface tension did not display any significant difference in lipid internalization (p-value of 0.21), despite the increased lipid fluidity observed upon surface tension (around 17% decrease in the order parameters, Figs 1D and S1). Thus, the main driving force for lipid internalization appears to be the charge of the lipid head group.

### Additional remark

It is worth noting that the simulations were initiated with a sodium ion in the allosteric sodium binding site [58] as we anticipated the possibility of observing sodium release during the conformational transition. In the majority of the simulations, the sodium ion remained in its allosteric site, as shown in S7A Fig. Nevertheless, under surface tension, the sodium ion transiently explored downward positions toward the intracellular side, reaching distances up to 10–12 Å from initial position. In two simulations, the sodium ion successfully escaped to the extracellular side. The sodium egress events were specifically observed in DOPG bilayers under surface tension. Interestingly, in one of these two cases, the presence of an internal DOPG molecule facilitated the escape of the sodium ion (see the mechanism in S7B Fig). These observations indicate that the sodium binding site is destabilized in our simulation conditions. Furthermore, they suggest that atypical or non-canonical conformations with an open TM7 might act as useful intermediates in receptor activation for facilitating sodium egress, which is a necessary step to complete the activation process [59].

## Discussion

Numerous experimental and computational data provide evidence that the AT1 receptor can exhibit different alternative and intermediate conformations, yielding a variety of cellular responses [31,40–44,60,61]. However, the precise mechanisms through which environmental conditions influence the conformational landscape of the AT1 receptor and, consequently, its functional diversity, remain poorly understood. Simulating the effects of physical factors in *in silico* simulations poses significant challenges, particularly in distinguishing between macro and micro-parameters that contribute to the observed effects. Nevertheless, these simulations are crucial for understanding, and potentially modifying, the responses of the AT1 receptor in various physiopathological conditions.

The steered method that we have developed offers a way to address this question by conducting multiple iterations under diverse environmental conditions, for a cumulative time of 1.9 μs by condition. The duration of each simulation is not long enough to achieve full receptor activation (see Results) or to inform on the long-term stability of the active state which has been investigated by others [31]. Nevertheless, during this duration, the receptor reaches intermediary states in the activation process which depend on the receptor physico-chemical environment. Thus, our steered method provides information on the *activation mechanism* of the AT1 receptor and the influence of environmental conditions on this process. This approach helps unravel the factors that depend on specific molecular interactions or on macroscopic physical parameters, providing insights into the mechanisms underlying the functional diversity of the AT1 receptor and its ability to work as a mechanosensor.

**Fig 6:**
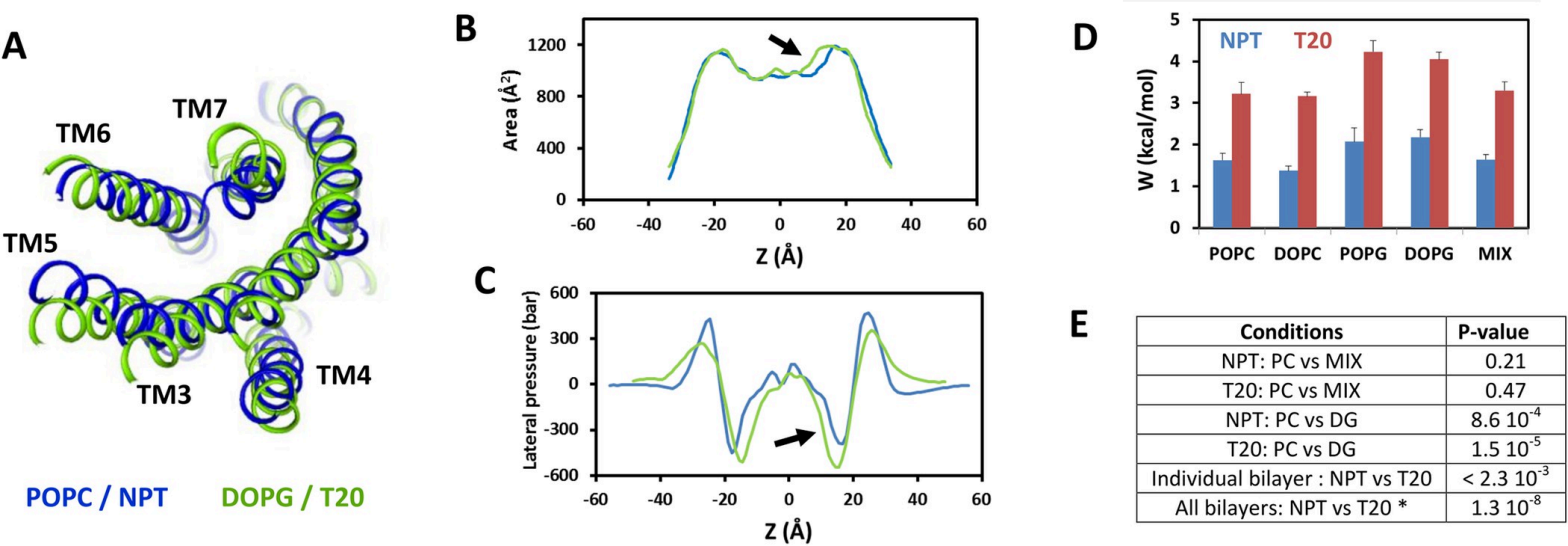
Impact of applied tension on the energetics of the receptor conformational change. (A) Superposition of the two conformations of AT1 obtained at the end of SMD simulations in POPC without applied surface tension (blue ribbon) and DOPG with applied surface tension (green ribbon) and presented in Fig 2. The receptors are viewed from the cytoplasm; (B) Cross-section areas of the AT1 receptor during the last 30 ns of the corresponding trajectories, with the same color code. In this example, the average area difference in the 0–20 Å range is 50 Å^2^, corresponding to a volume of 1000 Å^3^; (C) Lateral pressure profiles obtained in POPC without applied tension (blue) and in DOPG with applied tension (green), with embedded AT1 receptor; (D) Estimates of the work *W* against the pressure profile to shrink the receptor volume as shown in (B), according to Eq (1) and the lateral pressure profiles of each environment under scrutiny (Fig 1E). Data are average ± standard deviation of the three replicas carried out by condition; (E) Table giving the p-values for the probability of no significant difference (Null hypothesis) between data sets obtained in the indicated conditions. The p-values were calculated with the t-test, except for comparing the effect of the surface tension on pooled bilayers. In this latter case, p-values were calculated with the non-parametric Wilcoxon test (indicated by a star in the Table).

The simulation conditions used in our study have revealed a variety of AT1 conformations during the process of receptor activation, transitioning from an inactive state towards an active-like conformation. The position of TM7 is a straightforward criterion for defining these conformations (Fig 3). However, the conformations also differ in the positions of TM6 and other helices (Figs 3A, 4A, 6A, S3 and S4). Depending on the specific environmental conditions, these conformations can either participate in a continuum of intermediates in the activation process or remain stable over the duration of the simulations. One notable example is the atypical conformation, which is stabilized mainly when two environmental factors act simultaneously: (1) the presence of anionic lipids and (2) the application of surface tension. Comparison of the data obtained in five different lipid bilayers with and without surface tension may help understand factors important for the stabilization of the atypical conformation.

Environmental conditions do not modify the inactive conformation of the AT1 receptor observed during the preliminary classical MD simulations that we have performed (S2 Fig). This contrasts with our observations during the steered MD simulations, where the final conformations depend on environmental conditions (Figs 3 and 4). These observations indicate that during the process of conformational changes, the AT1 receptor “feels” its environment, which affects the energetics of the conformational changes. Kinetics and thermodynamics factors cannot be easily disentangled, since most parameters may affect both the free energy of the lipid-receptor system and the activation energy. Here, for the sake of clarity, we will focus on two factors: (1) the gross conformational change of the receptor, and (2) the reorganization of the lipid-receptor interactions.

The first factor depends on the applied surface tension. Indeed, when a protein is embedded into a membrane, changes in its cross-section area during a conformational transition make the transition sensitive to lateral pressure, because this change requires a work *W* done against the lateral pressure profile [62,63]. As a general rule, a conformation with increased cross-section area is favored by application of surface tension [62,63]. This effect can promote the opening of TM6 and receptor activation in response to stretching or shear stress, which has indeed been observed for the AT1 receptor [22–30]. We can evaluate the mechanical work necessary to modify the conformation of a receptor embedded in a membrane conformation. With the hypothesis that the change in protein conformation does not alter the pressure profile, the work *W* against the lateral pressure profile *π(z)* for a change *δA(z)* in the cross-section area is equal to [63]:

W=∫δA(z)π(z)dz (1)

To illustrate the impact of the gross conformation changes of the AT1 receptor in these simulations, we can consider the two limit cases shown in Fig 2 as examples of pre-active and atypical conformations. Several helices from TM3 to TM7 have a more open position in the atypical conformation than in the pre-active conformation (Fig 6A). As a consequence, the intracellular side of the AT1 receptor (*z* values in the 0–20 Å range) displays an increased cross-section area in the atypical conformation, as compared to the pre-active one (Fig 6B), which corresponds to a volume increase of around 1000 Å^3^. Thus, the work *W* for the transition from the atypical to the pre-active conformation strongly depends on the receptor environment through the lateral pressure profile *π(z)* (Fig 6C). Using Eq (1) and the pressure profiles in Fig 1E, we calculated the work required to shrink the receptor volume by 1000 Å^3^ according to Fig 6B, in the diverse environments. This work ranged from 1.4 kcal/mol in a DOPC bilayer without applied tension to 4.2 kcal/mol in a POPG bilayer under an applied tension of 20 dyn/cm (Fig 6D). Noteworthy, in these calculations, the work in the MIX bilayers was similar to the work in phosphocholine bilayers, in the same conditions of applied surface tension (Fig 6E). For each bilayer under scrutiny, an average increase of 1.8 ± 0.2 kcal/mol was observed upon application of the surface tension with p-values of 2.3 10^−3^ or less. When the bilayers were pooled, the p-value for comparing data with and without applied surface tension reached 1.3 10^−8^ with the Wilcoxon test. This effect should slow down the conformational transition, in the presence of surface tension. Nevertheless, the differences between neutral and anionic phospholipids (0.5 ± 0.1 kcal/mol and 0.9 ± 0.2 kcal/mol without and with applied tension, respectively) cannot explain alone the synergy between applied tension and anionic lipids to stabilize the atypical conformation, which is also observed in the MIX bilayer.

Specific interactions of the lipids with the receptor and reorganization of the lipids also contribute to the energetics of the transition. The H-bonds between the positively charged AT1 C-terminus and the lipid head groups are more frequent when the lipids are anionic (Fig 5A). This feature is frequently observed for GPCRs [12,64] and explains the increase concentration of anionic DOPG in the vicinity of the TM6-TM7-H8 cleft that we observed in the MIX conditions. These increased interactions also favor internalization of lipid molecules that is observed almost exclusively for anionic lipids, either in pure or heterogeneous bilayers (Fig 5C). Internal lipids and additional H-bonds observed with anionic lipids can prevent or slow down the inward motion of TM7 by steric hindrance or by imposing an additional energetic cost, thereby maintaining the receptor in non-canonical/atypical conformations. These additional costs might include disruption/reorganization of H-bonds or, in the work against the lateral pressure profile, the necessity to take into account the entire system formed by the receptor and interacting lipids. With a lipid area in the 60–70 Å^2^ range (Fig 1C), any additional lipid interaction with the AT1 receptor would markedly affect the energy penalty. These additional costs lead to a synergy between anionic lipids and applied surface tension to stabilize the atypical conformation, which we actually observe.

How can these findings relate to the AT1 receptor in physiological conditions? Biological membranes of the cardiovascular system are complex mixtures of phospholipids (including anionic phosphoserine, phosphatidyl acid and phosphatidylinositol), cholesterol, triglycerides and sphingolipids, with microdomains exhibiting specific compositions (lipid rafts). The precise composition depends on cell type, age, diet, health and disease, and is the subject of numerous lipidomics studies [18–21]. The pressure profile felt by a receptor thus depends both on the membrane composition and on mechanical stimuli, which can be local or transmitted from distant sources [65]. For AT1, mechanical stress involves stretching, for example in cardiomyocytes, and shear stress (blood flow), in blood vessels. As stretching, shear stress increases membrane fluidity [66,67] and lipid area [68], and thus should modify the lateral pressure profile. In addition, cholesterol modifies properties and lateral pressure profiles of biological membranes [69] and directly interacts with AT1 [70]. Cholesterol is thus an important component to be taken into account to mimic biological membranes. Further MD studies involving complex bilayers with cholesterol will allow decipher the AT1 response to mechanical factors, in environments closer to physiological (and pathological) conditions. Nevertheless, in spite of these limitations, our results may contribute to explain several observations.

The synergy between mechanical stress and anionic lipids that we have described might contribute to the capability of H8 to act as a sensor of shear stress in vascular arteries, which has been observed for AT1 and for the histamine receptor 1 (H1R), which also possesses a positively charged H8 [71]. The positions of the positive charges, which are not equivalent (Fig 5B), might allow differential responses between different receptors. The observed synergy might also explain the absence of consensus on the signaling pathways of AT1 activated by mechanical stress. Several studies suggest the activation of β-arrestin pathways by osmotic or mechanical stretch [23,24,27]. A study [23] proposes a distinct β-arrestin signaling pathway downstream of AT1 activated by osmotic stretch, compared to β-arrestin biased ligands. The authors report that an initial coupling of AT1 and Gαi induced by osmotic stretch is required for the recruitment of β-arrestin2 and activation of downstream pathways. Another study suggests that myogenic vasoconstriction, a fundamental mechanism aimed at regulating blood pressure and flow via mechanosensors in resistance arteries, is mediated in part by AT1 via Gq/11 without implication of angiotensin II [11]. Our study strongly suggests that, under mechanical stress, the local membrane composition may affect the mechanism of receptor activation, leading to diverse active conformations and thereby to distinct signaling pathways which would depend on the cellular context. This finding leads to an additional level of complexity in the mechanisms leading to AT1 (dys)regulation, which should be taken into account in drug design.

In summary, our results provide evidence for a multi-step process of AT1 activation that can be modified by specific environmental conditions, such as surface tension and the presence of anionic lipids. These external factors act as allosteric modulators of the activation process, potentially influencing both the rate of the transition and the final active conformation(s) of the receptor. Most importantly, surface tension and anionic lipids can act in synergy to stabilize an atypical AT1 conformation, characterized by outward position of both the TM6 and TM7 helices. Our results thus strongly suggest a role of anionic lipids in transmitting mechanical signals to AT1, which might in turn activate specific pathways. This is an important issue in biological context for understanding the development of diseases with impaired AT1 signaling in response to mechanical stress, such as chronic hypertension. Interestingly, anionic lipids preferentially bind the TM6-TM7-H8 cleft which corresponds to a putative allosteric site of AT1 [43]. Targeting this allosteric site in drug design may offer opportunities to develop allosteric ligands that selectively favor or hinder specific active conformations of the AT1 receptor. Reducing blood pressure remains challenging in a large number of hypertensive patients. A better control of myogenic tone could be obtained through the specific targeting of mechanosensitive receptors such as AT1, leading ultimately to a better control of blood pressure and, most importantly, of local blood flow to tissues.

## Methods

### Molecular modeling

Human AT1 was modeled from residues 17 to 317 as previously described [58], using the homology modeling software MODELER [72]. Modeling from residues 17 to 303 was based on the crystallographic structures of inactive AT1 (PDID: 4YAY [73] and 4ZUD [74]). The C-terminal part (residues 303 to 317) was modeled parallel to the membrane, using the inactive structure of the δ opioid receptor (OPRD) (PDB ID: 4N6H) [75] as a template. The choice of this template for the C-terminus is due to the absence of H8 in the 4ZUD structure and to a suspicious tilted orientation of H8 in the 4YAY structure, leading to a seesaw motion in MD simulations [76]. The selected orientation of H8 is corroborated by the structures of AT1 in active conformations (e. g. PDB ID: 6OS0, 6DO1, 7F6G). The missing parts of ECL2 and ICL3 were modelled with MODELER. The two disulfide bonds in the crystal structures (Cys18-Cys274 and Cys101-Cys180) were maintained in the model. Asp and Glu residues were negatively charged, Lys and Arg residues were positively charged and His residues were neutral. A sodium ion and seven water molecules were added to the model and positioned in the sodium binding cavity, by homology with OPRD. This allows the stability of negatively charged Asp74, located in the sodium binding cavity. There was no lipid modification of cysteines. We used the Ballesteros’ notation throughout the study with the following references: N46 (1.50), D74 (2.50), R126 (3.50), W153 (4.50), P207 (5.50), P255 (6.50), P299 (7.50). H8 residues were numbered by their distance from anchor residue 7.50 in TM7.

### Classical molecular dynamics simulations

The AT1 models were prepared for molecular dynamics simulations (MD) using the Charmm-Gui interface [77]. The models were embedded within different lipid bilayers with 60 lipids on each side, and solvated by aqueous layers which extended to 20 Å above protein limits, with a TIP3P model for water molecules with all atoms represented explicitly. The charges were neutralized by the addition of 150mM KCl. The bilayer systems with lipids alone were also prepared with the Charmm-Gui interface. They were composed of 60 lipids on each side, solvated by 20 Å aqueous layers, with charges neutralized by addition of 150 mM KCl.

Classical MD simulations (cMD) with no mechanical stress were carried out under NPT ensemble (constant number of molecules, pressure and temperature). cMD simulations under mechanical stress were carried out under the NPγT ensemble which differs from the previous one by the application of surface tension γ to the Newton equations of movement [47,78]. With the Z axis perpendicular to the membrane surface, the relation between the pressure *P*_*Z*_, perpendicular to the membrane, the pressure *P*_*T*_, tangent to the membrane, and the surface tension γ is given by:

$$\gamma =L_{Z}(P_{Z}-P_{T})$$ (2)

where *Lz* is the cell height. Stretching and compression correspond to positive and negative γ, respectively. The NPT ensemble ensures that the tangent and perpendicular pressures are equal and is equivalent to the NPγT ensemble with γ set to 0.

Molecular dynamics simulations were carried out using NAMD v2.13 MD software [79] and the CHARMM36 parameter set [80,81]. They were performed using the HPC resources of IDRIS, granted by GENCI (www.genci.fr). The equilibration of the systems was based on the default Charmm-Gui protocol. It includes: (1) an energy minimization step for 5000 iterations, to remove close contacts between atoms, (2) six MD steps in which harmonic restraints were gradually taken off to achieve a smooth relaxation, for a total of 1 ns, and (3) a 20 ns MD step carried out under the same conditions as the production run to achieve stable conditions. In the first two equilibration MD steps, the NVT ensemble at 310 K and time-step of 1 fs were used. The following equilibration and production steps were carried out at constant temperature (310 K), pressure (1 atmosphere), and, for MD simulations under mechanical stress, surface tension γ, using a 2 fs time-step for integration. The Particle Mesh Ewald method (PME) was used to calculate the electrostatic contribution to non-bonded interactions with a cutoff of 12 Å. The van der Waals interactions were progressively cut off from 10.0 to 12.0 Å. The SHAKE algorithm was applied to the system. In either NPT or NPγT ensembles, pressure was controlled by a modified Nosé-Hoover method in which Langevin dynamics was used to control fluctuations in the barostat [79]. In NPγT conditions, the surface tension γ was set to 20 dyn/cm, in order to obtain a lipid area stretch of about 10% (Fig 1C), which matches experimental data on mechanical activation of AT1 [27]. This value was well below the limit of stability of our system which crashed for applied surface tension larger than 50 dyn/cm. Each trajectory lasted 220 ns (20 ns for equilibration and 200 ns for production). The simulations for lipids alone were carried out in the same conditions, except for equilibration. In this latter case, the third equilibration step setting the system pressure required two rounds (the first one with 25 000 steps, the second one with 100 000 steps) instead of a single round of 125 000 steps, to insure system stability.

### Lateral pressure profile calculation

The lateral pressure profiles *π(z)* were measured from 3 replicas of 135 ns long classical MD simulations with the AT1 receptor embedded in the different bilayers, without and with applied surface tension of 20 dyn/cm. The simulation box was divided into 100 slabs (approximatively 1 Å thick), regularly spaced along the Z axis, perpendicular to the bilayer plane. The diagonal elements of the pressure tensor *p*_*xx*_*(z*, *t)*, *p*_*yy*_*(z*, *t)* and *p*_*zz*_*(z*, *t)* were measured at the center *z* of each slab, every 500 steps (equivalent to 1 ps), and recorded in the NAMD output file (NAMD pressureProfile option on).

At instant *t* and position *z*, the lateral pressure *π(z*, *t)* is defined as the difference between the normal and tangent components of the pressure tensor:

$$\pi (z,t)=\left(p_{xx}(z,t)+p_{yy}(z,t)\right)/2-p_{zz}(z,t)$$ (3)

In each replica, the lateral pressure profile *π(z)* was obtained from the time average of the π(z, t) values measured from 20 ns (to ensure the stability of the box) to the end of the 135 ns simulations, using an R script that (1) calculates raw *π(z*, *t)* from the diagonal elements of the pressure tensors and the cell height (measured with the PBCTools Plugin in VMD), (2) corrects the *z* position for the drift in the bilayer position (measured from the barycenter of the lipid phosphorus atoms with VMD), (3) averages corrected data over time, and (4) smooths the curve by averaging on a window of three consecutive points. Finally, in Fig 1E, we averaged the data from the 3 replicas carried out in the same environmental conditions.

### Steered molecular dynamics simulations

#### Quaternion formalism

The main characteristic of GPCR activation is the outward motion of TM6. To describe the activation mechanism, we developed a quaternion model for the opening of TM6 (Fig 2A). In this formalism, the rotation of a vector (here, the axis of the TM6 N-terminus) is described by (1) the rotation axis (u1, u2, u3) obtained from the vector product of the axes before and after the rotation and (2) the rotation angle *θ* around the (u1, u2, u3) axis. These parameters are condensed into the quaternion *Q*:

$$Q=(\cos(\theta /2),\sin(\theta /2)u_{1},\sin(\theta /2)u_{2},\sin(\theta /2)u_{3})$$ (4)

according to the formalism introduced by the mathematician WR Hamilton on 1843.

We developed a Perl script (S1 Script) to calculate the quaternion *Q* associated with the conformation transition from the PDB coordinates. This script was based on previous work on helix axis in proteins [45]. The reference residue to calculate the helix axes in the active and inactive conformations of AT1 was A6.39 in the middle of the N-terminus of TM6. Using our inactive AT1 model and the active 6OS0 AT1 structure [41], the reorientation of TM6 could be described by the quaternion *Q*_*orien*t_ = (0.953716951, 0.123092659825288, -0.271779496962083, 0.037524391849534). This quaternion corresponds to an angle of 35°.

#### Steered protocol

The quaternion formalism for steered MD simulations has been introduced in NAMD by Moradi and co-workers [46]. In our study, steered MD simulations were carried out with the application of two forces during 20 ns: (1) a pulling force applied on the N-terminal half of TM6 to change its orientation; (2) a restraint force applied to TM3 to maintain its original orientation. This latter force was mandatory to avoid the pivotal motion of the entire receptor upon application of the pulling force. In either case, the quaternion formalism was used. The maintained orientation of TM3 was described by the (1,0,0,0) quaternion, as initial and final targets. The opening motion of TM6 from residues 6.32 to 6.48 was described by a reorientation from the initial orientation (1,0,0,0) to the target orientation described by *Q*_*orient*_. The same pulling force was applied in both cases and was equal to 5000 kcal/(mol * rad^2^) ≈ 1.52 kcal/(mol * deg^2^). The pulling velocity was set for TM6 to fulfil the opening motion within a timeframe of 20 ns (see Fig 2B). After the pulling force was applied for duration of 20 ns, the simulations continued without any restraint by classical MD simulations for 70 ns to ensure that the AT1 receptor reaches a relaxed conformation around the open TM6 conformation. The SMD simulations were initiated from snapshots obtained by classical MD simulations in the same conditions (NPT or NPγT ensembles). As the velocities and the xsc files were necessary to start the SMD simulations, we used the restart files from the cMD simulations that were saved every 20ns in our standard cMD procedure. Due to receptor breathing in cMD simulations, in each condition, we selected three snapshots after visual inspection to ensure that the starting conformation was “strictly” inactive, and then we launched replicas for steered simulations. In most cases, the steered simulations resulted in straight TM6, but, in about 20% of the attempts, TM6 became distorted during the simulations. In that case, the replica was excluded for final analysis and a new replica was launched. The simulations were reiterated up to obtain 7 replicas suitable for analysis from each initial snapshot. For easy visualization of the receptor conformational changes during the simulations, for each replica, a merged pdb file containing 12 equidistant snapshots obtained during the 90 ns timeframe of the SMD simulations has been deposited at the Mendeley data repository (doi: 10.17632/j4kst5nwk4.1).

### MD analysis

Analyses of the simulations were carried out with home-developed scripts using either the tcl language and utilities from VMD [82] or the R language and utilities from Bio3D [83]. Graphical analyses were carried out with VMD or with Pymol (https://pymol.org/). Distances between TM3 and TM6 and between TM3 and TM7 were calculated with Bio3D, by measuring the distances between the Cα atoms of residue 3.50 and either residue 6.34 or residue 7.55. Heatmaps were built with the Complex heatmap package [84]. The analysis of the H-bonds between lipids and the receptor and the search of internal lipids were carried out with VMD. We measured H-bonds between lipids and residues 7.58, 7.59, 7.61 and R7.62 in AT1. The cutoffs selected for the H-bonds were 3.5 Å for the distance and 30° for the angle. For internal lipids, we measured the distances between each phosphorus atom of the lipids in the intracellular leaflet and residues 3.50 and 7.56. Lipids were considered as internal when the following conditions were fulfilled: distance to TM3 was lower than 15 Å, distance to TM7 was lower than 12 Å and each one of these distances were lower than the distance between residues 3.50 and 7.56. This latter condition was necessary to exclude external lipids at the vicinity of the TM5-TM6 cleft.

Cross-sections of AT1 were measured in 15 snapshots regularly spaced in the final 30 ns of each SMD trajectory. The snapshots were superimposed on the Charmm oriented, initial model of AT1, and then cross-section areas were measured using the script provided by Charmm-Gui (step2.inp in the Charmm-Gui procedure [77]), and run locally using Charmm (c48b1 version). The .plo output files from Charmm, which give the cross-section areas of the protein at each 0.2 Å along the Z axis, were gathered in a single.csv file using a bash script for subsequent analysis with R. Data were averaged over the 15 cross-sections measured for each replica. Finally, we summarized the data by the averaged value of the cross-section areas in the 0 to 20 Å range. This range corresponds to the receptor moiety which is embedded into the intracellular bilayer leaflet and differs between receptor conformations (Figs 6A and 6B).

### Lipid properties

Lipid properties were measured in classical MD simulations. Membrane thickness was analyzed with MEMBPLUGIN [85] in VMD, both in the absence and in the presence of embedded AT1 receptor. Bilayer thickness was calculated from the peak to peak distance of the mass distribution of the lipid phosphorus atoms. In the absence of the receptor, the area per lipid was calculated either crudely from the cell size with the PBCTools plugin in VMD or by Voronoi analysis, using MEMBPLUGIN, without significant differences. In the presence of the receptor, the area per lipid was estimated from the cell surface to which was subtracted the cross-section area of the receptor (determined by the average of the top and bottom areas measured with Charmm).

The order parameters were computed with MEMBPLUGIN from the orientation *θ* of the C-H bond vectors with respect to the bilayer normal (here the Z axis of the system) averaged over all the lipids and the simulation times (represented by 225 snapshots).


$$S_{CH}=<3{\left(\cos\theta \right)}^{2}-1>/2$$ (5)

### Statistical analysis

The statistical analysis for distances and cross-section areas, which have normal distributions, were carried out with the Student’s t-test. The statistical analysis for H-bonds and lipid internalization, which have very spread distributions, were carried out with the non-parametric Wilcoxon test (rank-sum test). The statistical analysis of the work *W* against lateral pressure was carried out with the Student’s t-test with the hypothesis of normal distribution. The only exception was for the data from all the bilayers under scrutiny pooled by the applied surface tension. In that case, both with and without applied tension, the distribution of the data was clearly bimodal and we used the Wilcoxon test for the statistical analysis.

## Supporting information

S1 Fig Order parameters of the aliphatic chains of DOPC, DOPG, POPC and POPG during classical MD simulations. (TIF)

S2 Fig Impact of environmental conditions on the inactive AT1 receptor conformation investigated by classical MD simulations. (TIF)

S3 Fig 2D plots of the time evolution of the AT1 conformation during steered MD simulations. (TIF)

S4 Fig Time evolution of the TM3-TM6 distances during steered MD simulations of the AT1 receptor in diverse environments. (TIF)

S5 Fig Cross-section areas of the AT1 conformations obtained by steered MD simulations in diverse environments. (TIF)

S6 Fig Time evolution of the H-bond interactions between the lipids and the receptor C-terminus (K7.58, K7.59, K7.61 and R7.62) during steered MD simulations in diverse environments. (TIF)

S7 Fig Sodium behavior during steered MD simulations. (TIF)

S1 Script Script for quaternion calculation. (PDF)
