# Supplementary figures and images for: Mechanical stress and anionic lipids synergistically stabilize an atypical structure of the angiotensin II type 1 receptor (AT1)
Source: PLoS Comput Biol. 2024 Nov 13;20(11):e1012559. doi: 10.1371/journal.pcbi.1012559 (PMC11560033; doi:10.1371/journal.pcbi.1012559)

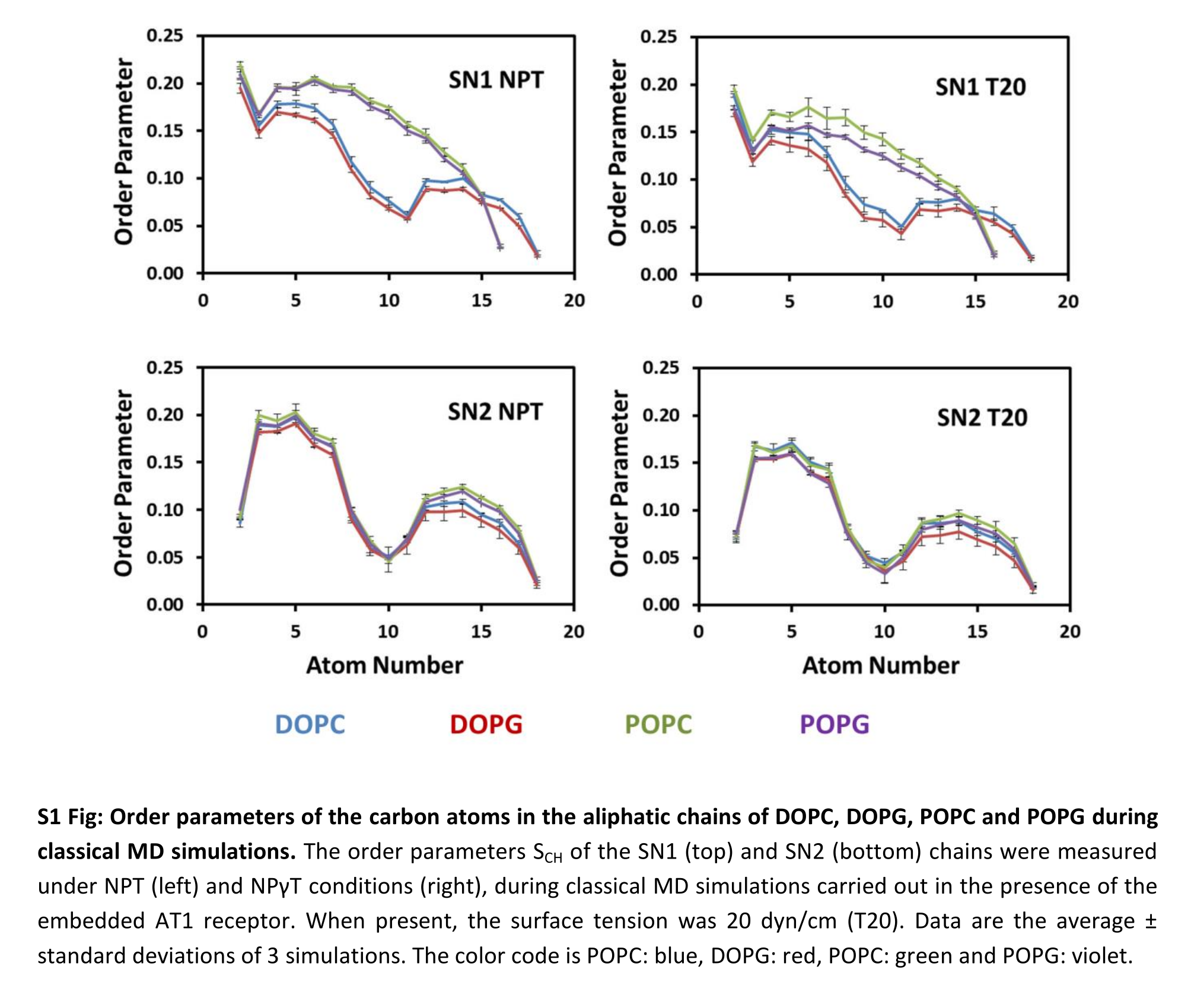

Supplement: S1 Fig — (TIF) [file pcbi.1012559.s001.tif]

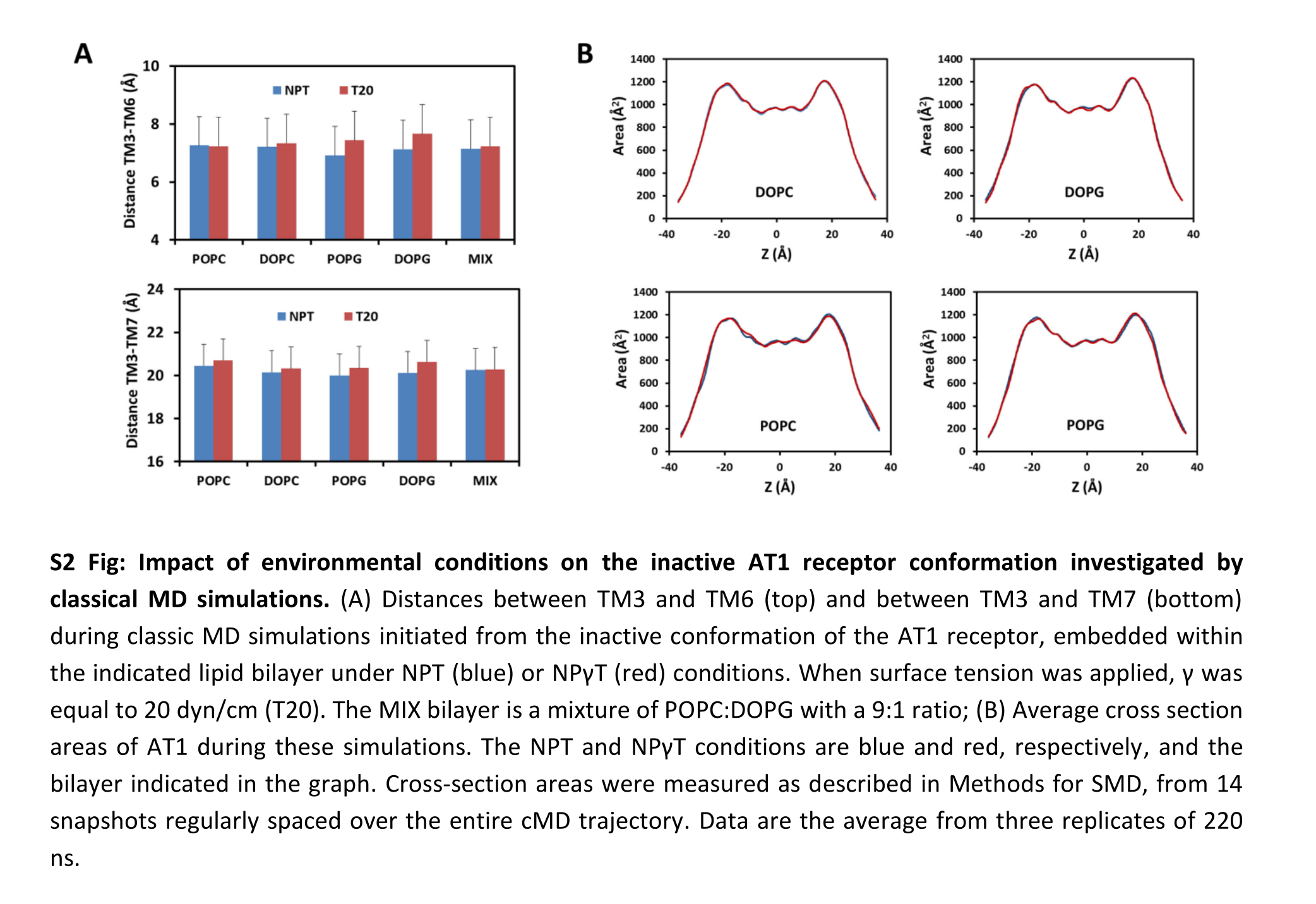

Supplement: S2 Fig — (TIF) [file pcbi.1012559.s002.tif]

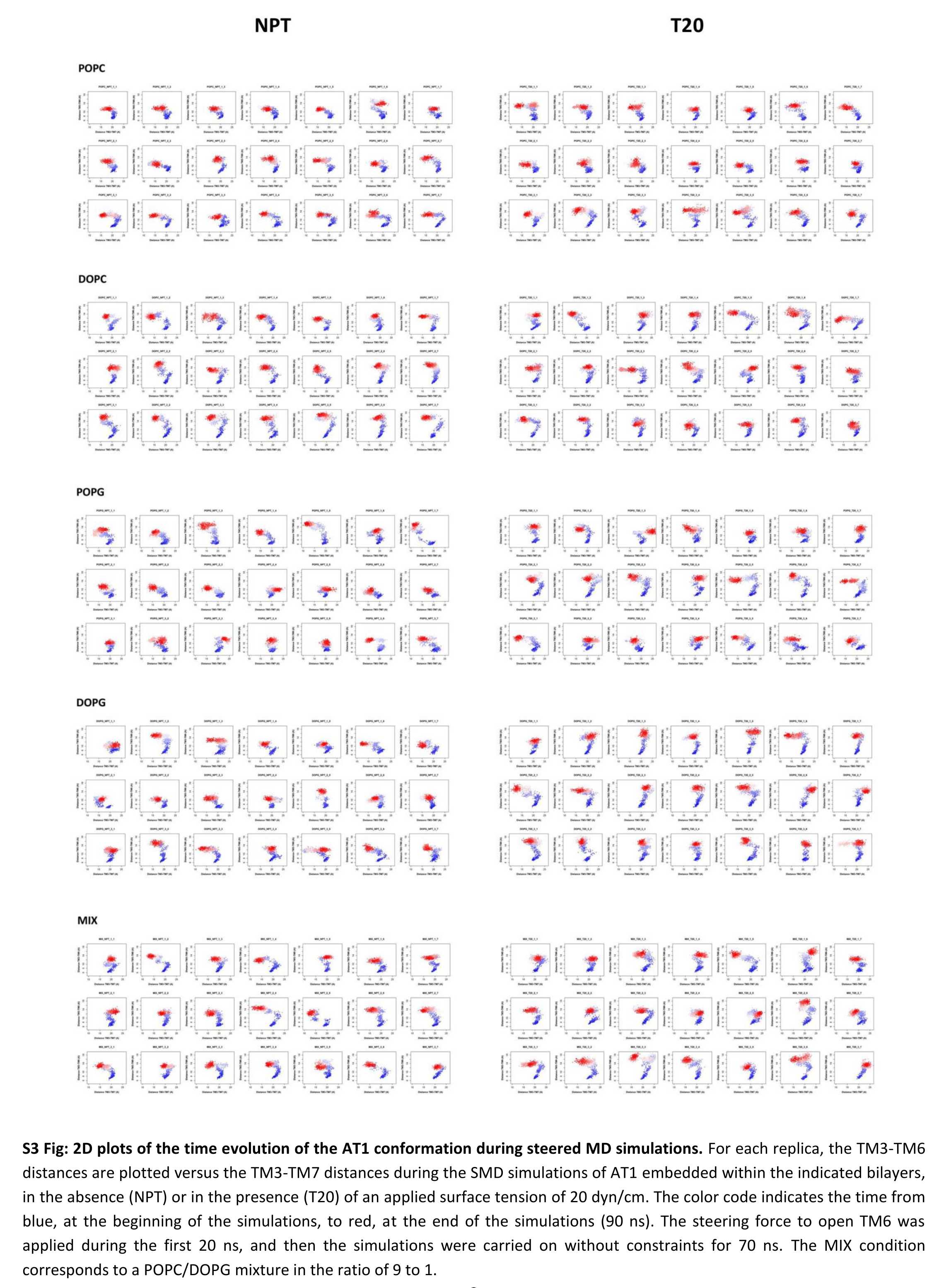

Supplement: S3 Fig — (TIF) [file pcbi.1012559.s003.tif]

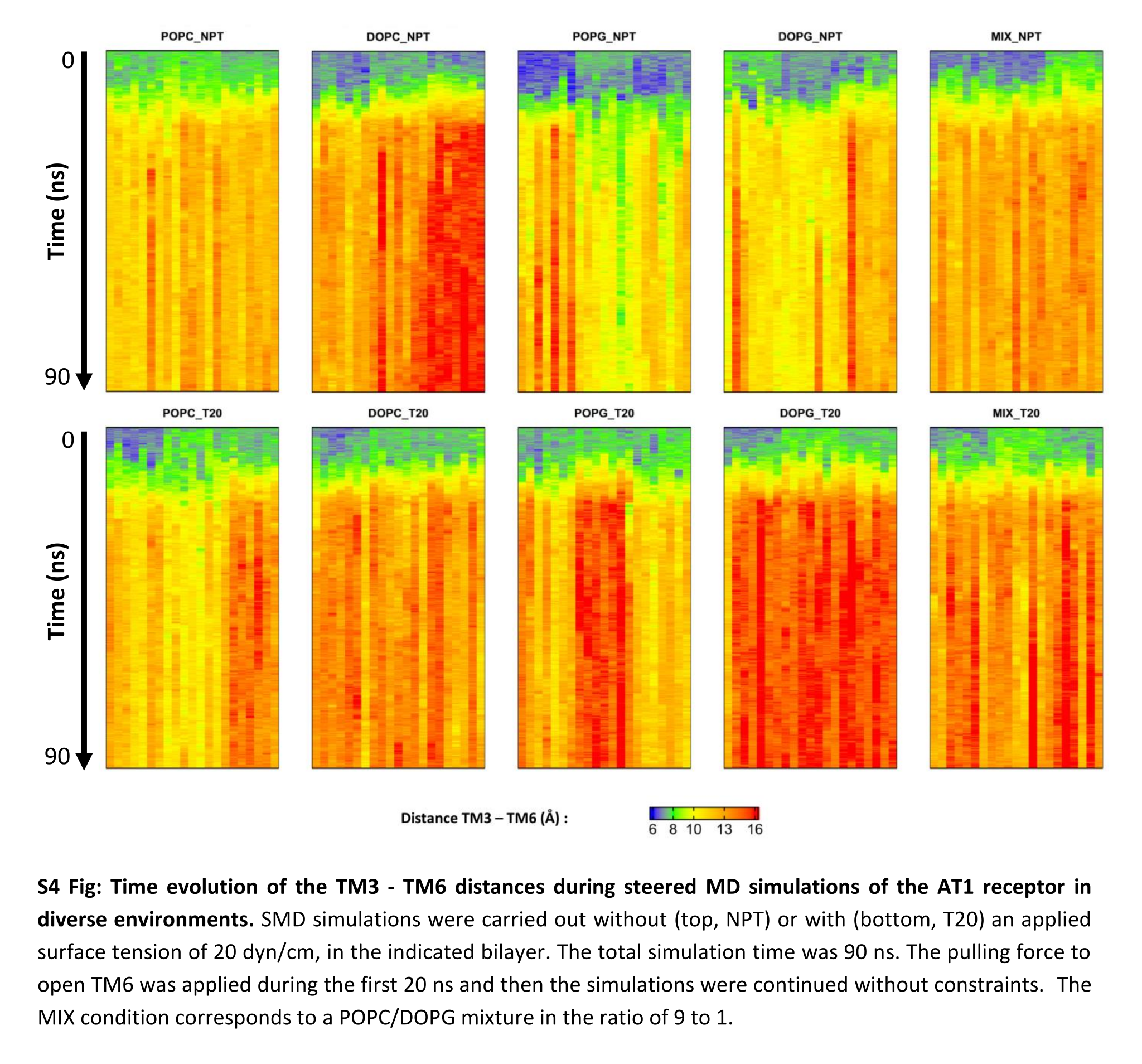

Supplement: S4 Fig — (TIF) [file pcbi.1012559.s004.tif]

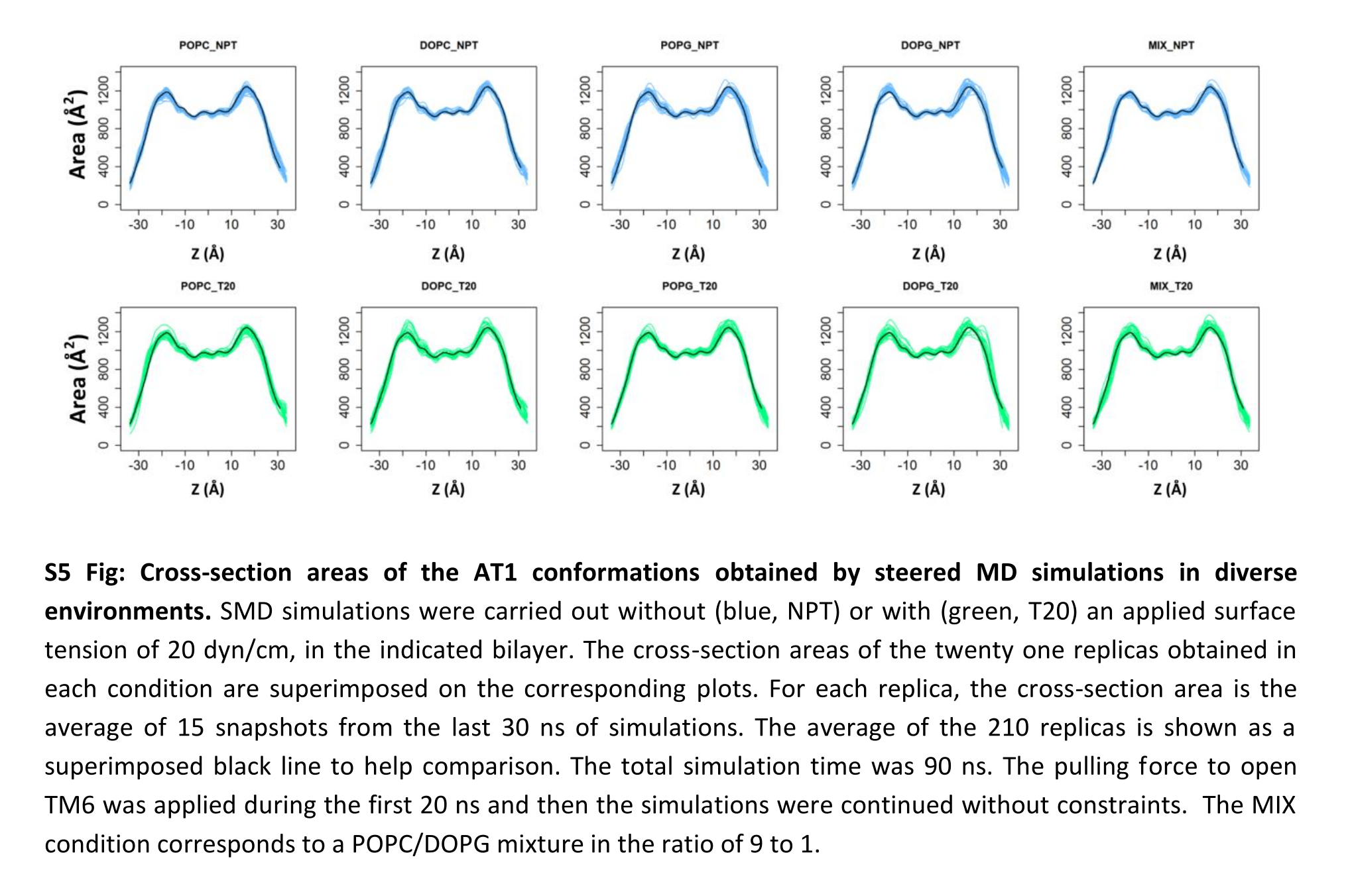

Supplement: S5 Fig — (TIF) [file pcbi.1012559.s005.tif]

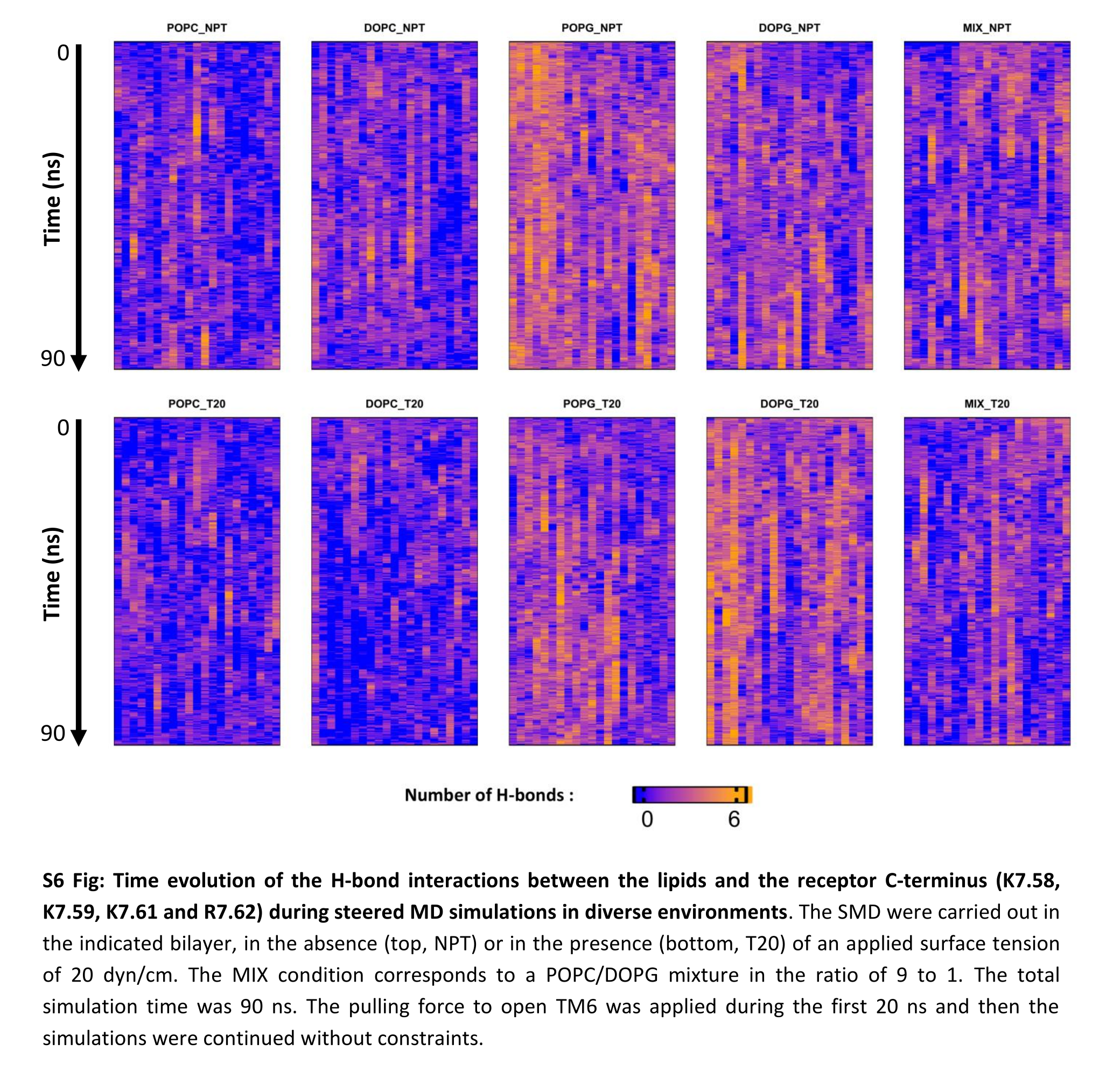

Supplement: S6 Fig — (TIF) [file pcbi.1012559.s006.tif]

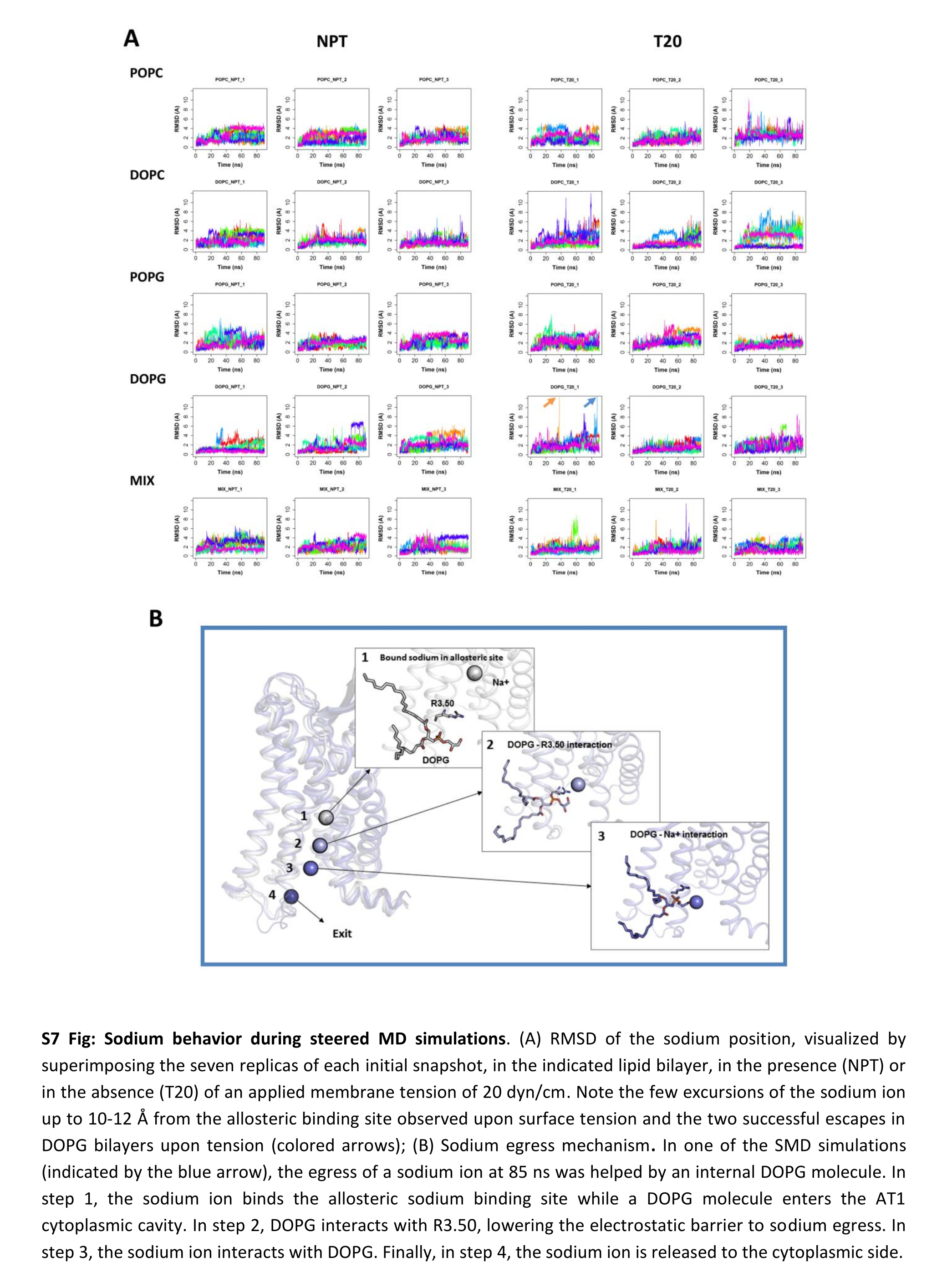

Supplement: S7 Fig — (TIF) [file pcbi.1012559.s007.tif]
