# Supplementary material for: Mechanical stress and anionic lipids synergistically stabilize an atypical structure of the angiotensin II type 1 receptor (AT1)
Source: PLoS Comput Biol. 2024 Nov 13;20(11):e1012559. doi: 10.1371/journal.pcbi.1012559 (PMC11560033; doi:10.1371/journal.pcbi.1012559)
Supplement: S1 Script — (PDF) [file pcbi.1012559.s008.pdf]

S1 Script: quaternion.pl

```
#! /usr/bin/perl
#############################################################
# Compute reorientational quaternion between two helices
# from initial orientation described by the (1,0,0,0) quaternion
#############################################################

use strict;
use Math::Trig;

my $pdb_ref;
my $pdb_act;
my $aa_ref;
my $aa_act;

my $angle_rot;
my $half_rot;
my $rot_deg;
my $half_deg;

my @coord_ref;
my @coord_act;
my @axis_ref;
my @axis_act;
my @axis_rot;
my @quaternion;

$pdb_ref ="my_pbd_ref.pdb";  # Reference structure oriented with charmm-gui
$pdb_act ="my_pdb_act.pdb";  # Active structure superposed on the reference structure
$aa_ref = n1;        # first residue of segment to calculate reference axis
$aa_act = n2;        # first residue of segment to calculate active axis (MUST BE SAME RESIDUE IN STRUCTURE but may have different numbering)

@coord_ref = coord_helix($pdb_ref,$aa_ref);
@coord_act = coord_helix($pdb_act,$aa_act);

@axis_ref = axis_helix(@coord_ref);
@axis_act = axis_helix(@coord_act);

@axis_rot = produit_vectoriel(@axis_ref, @axis_act);
@axis_rot = normalisation(@axis_rot);

$angle_rot = angle_entre_vecteurs(@axis_ref, @axis_act);
$rot_deg = ($angle_rot/pi)*180;

$half_rot = $angle_rot/2;
$half_deg = ($half_rot/pi)*180;

@quaternion = (cos$half_rot, sin$half_rot*$axis_rot[0], sin$half_rot*$axis_rot[1], sin$half_rot*$axis_rot[2]);
print "Coordonnées du quaternion \n";
print "@quaternion \n";



############################################################################
# Reads PDB and
# return a table with the coordiantes of the 4 CA that define the helix axis
############################################################################

sub coord_helix{
          my ($file, $aa_number) = @_;
          my @coord = ();
          open (FILE,"$file") or return 0;
          print "$file\n";
          print "$aa_number\n";

          while (my $line = <FILE>){

                    if ($line =~ /ATOM/) {
                              my $atom = substr $line, 12, 4;
                              my $res_number = substr $line, 22, 4; #columns 23-26
                              my $x = substr $line, 30, 8; #columns 31-38
                              my $y = substr $line, 38, 8; #columns 39-46
                              my $z = substr $line, 46, 8; #columns 47-54
                              $atom =~ s/\b\s//;
                              $res_number =~ s/\b\s//;
                              $x =~ s/\b\s//;
                              $y =~ s/\b\s//;
                              $z =~ s/\b\s//;

                              my $ind = 0;
                              while ($ind < 4){

                                        if (($atom =~/CA/) && ($res_number == $aa_number + $ind))  {
                                                  print ($atom, $res_number, $x, $y, $z, "\n");
                                                  push @coord, $x, $y, $z;
                                        }
                              $ind += 1;
                              }
                    }
          }
          close(FILE);
          return(@coord);

}

############################################################################
# vecteur
# Creates vector
############################################################################

sub vecteur {
          my ($xa, $ya, $za, $xb, $yb, $zb) = @_;
          return ( ($xb-$xa), ($yb-$ya), ($zb-$za) );
}
```

```perl
################################################################################
# norme
# returns the norm of a vector
################################################################################

sub norme {
	my ($x, $y, $z) = @_;
	return ( sqrt( ($x*$x) + ($y*$y) + ($z*$z) ) );
}


################################################################################
# normalisation
# normalizes a vector
################################################################################

sub normalisation {
	my ($x, $y, $z) = @_;
	return ( ( $x / norme($x, $y, $z) ), ( $y / norme($x, $y, $z) ), ( $z / norme($x, $y, $z) ) ) unless (norme($x, $y, $z) == 0);
}


################################################################################
# produit scalaire
# returns the scalar product of two vectors u and v
################################################################################

sub produit_scalaire {
	my ($xu, $yu, $zu, $xv, $yv, $zv) = @_;
	return ( ($xu*$xv) + ($yu*$yv) + ($zu*$zv) );
}


################################################################################
# produit vectoriel
# returns the cross product of two vectors u et v
################################################################################

sub produit_vectoriel {
	my ($xu, $yu, $zu, $xv, $yv, $zv) = @_;
	return ( (($yu*$zv)-($zu*$yv)), (($zu*$xv)-($xu*$zv)), (($xu*$yv)-($yu*$xv)) );
}


################################################################################
# angle entre vecteurs
# returns the angle formed by two vectors u and v
################################################################################

sub angle_entre_vecteurs {
	use Math::Trig;
	my ($xu, $yu, $zu, $xv, $yv, $zv) = @_;
	return ( acos( produit_scalaire($xu, $yu, $zu, $xv, $yv, $zv) / ( norme($xu, $yu, $zu) * norme($xv, $yv, $zv) ) ) );
}


####################################################################################
# returns the vector in the plane of the helix.
# The vector in the plane is obtained from the positions of the CA1, CA2 and CA3
# atoms of the helix. It points towards the helix center if positioned at CA2
####################################################################################

sub vecteur_plan_helice {
	my ($xu, $yu, $zu, $xv, $yv, $zv, $xg, $yg, $zg) = @_;
	# Compute i
	my ($xi, $yi, $zi) = vecteur ($xu, $yu, $zu, $xv, $yv, $zv);
	($xi, $yi, $zi) = normalisation ($xi, $yi, $zi);
	# Compute j
	my ($xj, $yj, $zj) = vecteur ($xv, $yv, $zv, $xg, $yg, $zg);
	($xj, $yj, $zj) = normalisation ($xj, $yj, $zj);
	# Compute cross product of i and j
	my ($xw, $yw, $zw) = produit_vectoriel ($xi, $yi, $zi, $xj, $yj, $zj);
	($xw, $yw, $zw) = normalisation ($xw, $yw, $zw);
	# Compute cross product of i and w
	my ($xt, $yt, $zt) = produit_vectoriel ($xi, $yi, $zi, $xw, $yw, $zw);
	# Compute angle between 2 vectors i and j
	my $theta = angle_entre_vecteurs ($xi, $yi, $zi, $xj, $yj, $zj);
	# Compute vector k that points towards the center of the helix
	my $xk = ((cos($theta/2))*$xi)+((sin($theta/2))*($xt));
	my $yk = ((cos($theta/2))*$yi)+((sin($theta/2))*($yt));
	my $zk = ((cos($theta/2))*$zi)+((sin($theta/2))*($zt));
	return ($xk, $yk, $zk);
}

################################################################################
# axis_helix
# returns the vector of the axis of the helix
# from coordinates of CA1,CA2,CA3 and of CA2,CA3,CA4.
# In each case, a vector in the plane of the three CA is computed
# then the cross product of these two vectors will give the helix axis
################################################################################

sub axis_helix {
	use strict;
	use Math::Trig;
	my @coord = @_;
	# Compute vector k perpendiculat to the helix at n
	my ($xk, $yk, $zk) = vecteur_plan_helice ($coord[0], $coord[1], $coord[2], $coord[3], $coord[4], $coord[5], $coord[6], $coord[7],
$coord[8]);
	# Compute kp vector perpendicular to the helix at n+1
	my ($xkp, $ykp, $zkp) = vecteur_plan_helice ($coord[3], $coord[4], $coord[5], $coord[6], $coord[7], $coord[8], $coord[9], $coord[10],
$coord[11]);
	# computate the cross product of k and kp to obtain the axis of helix
	my ($xu, $yu, $zu) = produit_vectoriel ($xk, $yk, $zk, $xkp, $ykp, $zkp);
	($xu, $yu, $zu) = normalisation ($xu, $yu, $zu);
	return ($xu, $yu, $zu);
}
```
